# Residence Near Agricultural Crops at Birth and Risk of Adult Testicular Germ Cell Tumors: A French Nationwide Case–Control Study Using Historical Aerial GIS Data

**DOI:** 10.1002/ijc.70469

**Published:** 2026-05-18

**Authors:** Aurélie M. N. Danjou, Astrid Coste, Lény Grassot, Elodie Faure, Carlos Crispim‐Junior, Laure Tougne Rodet, Olivia Pérol, Rémi Béranger, Jeanne Perrin, Barbara Charbotel, Joachim Schüz, Béatrice Fervers, the TESTIS study group, Helen Boyle, Aude Fléchon, Elodie Belladame, Véronique Drouineaud, Céline Chalas, Myriam Virlouvet, Lucile Ferreux, Guillemette Perier, Pauline Peretout, Diane Rivet, Sandrine Rulle, Florence Eustache, Nathalie Sermondade, Marine Durand, Rachel Levy, Isabelle Berthaut, Jacqueline Saias‐Magnan, Catherine Metzler‐Guillemain, Carole Daoud‐Deveze, Louis Bujan, Myriam Daudin, Florence Brugnon, Valérie Bruhat, Cyril Bouche, Isabelle Kocsinski, Marius Teletin, Françoise Schmitt, Nathalie Guyomard, Aline Papaxanthos, Volcy Soula, Sandrine Giscard d’Estaing, Pascale Dehee, Delphine Yalcinkaya, Céline Bouillon, Cynthia Frapsauce, Anne Viallon, Bérengère Ducrocq, Marie‐Ange Clarotti, Ethel Szerman, Amélie Ancelle, Oxana Blagosklonov, Séverine Bey, Célia Ravel, Ségolène Veau, Agnès Letremy, Patricia Fauque, Julie Barberet, Stéphanie Lattes, Emmanuelle Thibault, Catherine Diligent, Christel Hersant, Vanessa Loup‐Cabaniols, Anna Gala, Sylvianne Hennebicq, Julien Bessonnat, Marie‐Claude Blocquaux, Brigitte Dananché, Johan Spinosi, Matthieu Dubuis, Charlotte Carretero, Etienne Durand, Bénédicte Thomas, Rémi Ratajczak

**Affiliations:** ^1^ Environment and Lifestyle Epidemiology Branch, International Agency for Research on Cancer/World Health Organization Lyon France; ^2^ Departement Prévention, Cancer et Environnement Centre Léon Bérard Lyon France; ^3^ Inserm U1296 Radiation: Defense, Health, Environment Lyon France; ^4^ University Paris‐Saclay, UVSQ, Inserm, Gustave Roussy, “Exposome and Heredity” Team, CESP UMR1018 Villejuif France; ^5^ Univ Lyon 2, CNRS, INSA Lyon, UCBL, Centrale Lyon, LIRIS Bron France; ^6^ Univ Rennes, CHU Rennes, Inserm, EHESP, Irset (Institut de Recherche en Santé, Environnement et Travail), UMR_S 1085 Rennes France; ^7^ Fédération Française des CECOS Paris France; ^8^ CNRS, IRD, IMBE Avignon University, Aix Marseille University Marseille France; ^9^ Centre Clinico‐Biologique d’AMP‐CECOS AP‐HM La Conception University Hospital Marseille France; ^10^ UMRESTTE, UMR T 9405, Ifsttar, Lyon 1 University, Lyon University, Eiffel University Bron France

**Keywords:** agricultural farmland, case–control study, geographic information system, pesticides, testicular germ cell tumors

## Abstract

Early life exposure to pesticides, in particular through pesticides drift in residents nearby agricultural crops, is suspected to increase testicular germ cell tumors (TGCT) risk. We conducted a hospital‐based case–control study of 472 TGCT cases and 683 matched controls aged 18–45 years in France and estimated adult TGCT risk associated with agricultural surfaces around residence as a surrogate for environmental exposure to agricultural pesticides at time of birth. Surface of arable lands, orchards and vineyards in 500 m buffer around the participants’ residential address at birth was assessed using geographic information system (GIS) methods and software semi‐automatic processing of historical aerial images. Odds ratios (OR) for TGCT (overall and by histological subtype) and 95% confidence intervals (CI) were estimated using conditional logistic regression. No increased risk of TGCT was observed for presence of arable lands (OR = 1.14, CI = 0.86–1.52), vineyards (OR = 0.88, CI = 0.56–1.36) and agricultural surfaces overall (OR = 1.13, CI = 0.84–1.51) in 500 m buffer around residence at birth. Presence of orchards (prevalence: 8.7%) was associated with a modest increase in TGCT risk (OR = 1.51, 95% CI = 0.99–2.32), slightly stronger for seminoma (OR = 1.78, 1.07–2.97) and in participants conceived or in their first trimester after conception during spring–summer (OR = 1.70, 1.03–2.82). In conclusion, the study found no overall association between adult TGCT risk and agricultural crops around residence at birth, except for the presence of orchards, with a possible increase in seminoma risks, as well as TGCT risk in participants for whom the first trimester after conception fell into spring–summer.

## Abstract (highlights)

What's new?

As the incidence of testicular germ cell tumors (TGCTs) rises, prenatal exposure to agricultural pesticides may represent an important risk factor. Here, geographic information system‐based measures of farmland proximity at birth were used as a proxy for early‐life pesticide exposure in France, a major pesticide consumer. Overall, TGCT risk was unrelated to proximity to most agricultural land types. However, residence near orchards was linked to a modestly elevated risk, particularly for seminoma and for those whose first trimester occurred in spring‐summer. The findings suggest a limited but specific association, warranting further investigation of critical pesticide exposure windows and TGCT risk.

## Graphical abstract

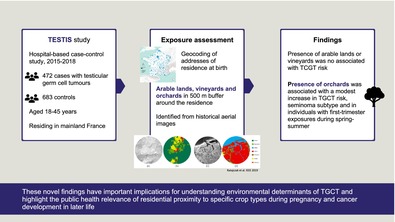

**Abbreviations**
CECOS: *Centres d’étude et de conservation des oeufs et du sperme*
CI: confidence interval
GCNIS: germ cell neoplasia in situ
GIS: Geographic Information System
IARC: International Agency for Research on Cancer
ICD‐O: International Classification of Disease for Oncology
IGN: French National Institute of Geographic and Forest Information
ISCO: International Standard Classification of Occupations
NAF: *Nomenclature d’activités française*
OR: odds ratio
PCS: professions and socio‐professional categories
SAS: Statistical Analysis System
TGCT: testicular germ cell tumors
WHO: World Health Organization

## 1 Introduction

Testicular germ cell tumors (TGCT) are rare tumors but represent the most common solid malignancy in men aged 15–44 years, and mainly affect Caucasian populations in high‐income countries. The incidence rate has been rising worldwide for the past four decades [1, 2], and the 2800 new cases estimated in France in 2018 have already exceeded the numbers predicted for 2025 [3, 4]. TGCT derive from germ cell neoplasia in situ (GCNIS) and comprise two main histological subtypes, seminomas and non‐seminomas, the latter also including mixed tumors [5].

Genetic predispositions and history of testicular cancer have been established as TGCT risk factors but cannot alone explain these trends. Geographical and temporal changes in incidence rates between population groups and regions, as well as in migrant populations, support an environmental origin of TGCT, and given the early onset of TGCT in life, prenatal and perinatal factors are putative TGCT risk factors [6]. Moreover, environmental exposures of the fetal testis are thought to result in testicular dysgenesis involving congenital male reproductive disorders (cryptorchidism, hypospadias, impaired spermatogenesis, and TGCT) [7].

Endocrine disrupting chemicals such as pesticides are suspected to be involved in the occurrence of TGCT [8], in particular during the mother’s pregnancy and early infancy, which are critical periods of genital organs’ development, in particular the first trimester of pregnancy that has been identified as the masculinization programming window [9]. France is the leading agricultural producing country in Europe and the second largest consumer of pesticides for agriculture [10]. Apple orchards, in particular, represent some of France’s most heavily sprayed agricultural lands, with frequent fungicide applications—especially from March to August [11]. Although it has been decreasing over the last three decades, the annual average pesticide use in France remains well above that of Europe and the World [12].

Positive associations for TGCT and subtypes have been observed recently with pre‐ and postnatal maternal exposure to organochlorines [13], or prenatal residential proximity to agricultural insecticides (organophosphates and carbamates) in adolescents aged 15–19 years old [14]. There is growing evidence of increased pesticide exposure and related adverse health effects among non‐farming residents living in the proximity of pesticide treated lands as a result of pesticide drift from sites of application [15, 16]. A study also reported correlations between residential proximity to crops in buffers of several sizes (100–750 m), crop acreage and detection of herbicides in indoor dust [17]. Using data from a French nationwide case–control study, we observed increased risks of adult TGCT following prenatal domestic usages of fungicides by the mother [18] and occupation as agricultural workers [19, 20].

This research aimed at estimating the risk of adult TGCT and subtypes associated with geographic information system (GIS)‐based estimates of cultivated farmland proximity to residence at birth as a spatial surrogate of exposure to agricultural pesticides at time of birth.

## 2 Methods

### 2.1 Study Design

We analysed data from a 3‐year prospective case–control study conducted in 20 out of the 23 university hospital centers in mainland France [18, 21]. Participants born in mainland France were eligible. Cases were male patients aged 18 to 45 years and newly diagnosed (time to diagnosis < 12 months) with primary GCNIS‐related TGCT between 2015 and 2018 and referred to the regional sperm banks located in the university hospitals for semen preservation prior to treatment (*Fédération Française des Centres d’étude et de conservation des oeufs et du sperme*, CECOS).

TGCT cases were histologically validated by reviewing the pathology reports and serum tumor markers (84%) by a TGCT expert. Using the International Classification of Disease for Oncology (ICD‐O) and the World Health Organization (WHO) classification of tumors of the urinary system and male genital organs, TGCT were classified as seminomas (9061/3) or non‐seminomas (embryonal carcinoma, 9070/3; choriocarcinoma, 9100/3; post‐pubertal yolk sac tumor, 9071/3; post‐pubertal teratoma, 9080/3; and mixed germ cell tumor, 9085/3) [5, 22]. One case of regressed germ cell tumor (9080/1) was not considered as seminoma or non‐seminoma. The proportion of false‐positive TGCT being low (5.1%), 43 TGCT patients (7.8%) not confirmed due to missing pathology reports were included as cases in the analyses.

Two groups of controls free of cryptorchidism and testicular cancer were included, across mainland France. Group A controls were recruited in CECOS among sperm donors or in assisted reproduction treatment centers among partners (with normal sperm production) of women undergoing examinations for fertility disorders. Group B controls were recruited in specialized maternity wards (equipped with maternal and neonatal intensive care units) among partners of women treated for a pathological pregnancy. Controls were frequency‐matched to cases on year of birth (5‐year categories) and hospital centres’ administrative region.

### 2.2 Data Collection

A structured questionnaire was administrated over the phone by trained interviewers from the Ipsos Company, blinded to the case–control status, to collect data from the study participants and their mothers/relatives [21]. Residential history from birth onwards was reported, including detailed information on each address, such as periods of residence, street name and number, postal code, locality, and any complement and remarkable point close to the home. Data collected also included occupational history, socioeconomic factors, birth characteristics, medical history, and lifestyle factors.

The mothers/relatives provided additional data covering pregnancy and postnatal periods (treatments, age, morphology, breastfeeding), their occupational history and that of the father, their residential history and their domestic use of pesticides (for pest control, gardening, against mold, on plants and pets) at several key points in time including during pregnancy and birth [18]. All interviewees were compensated after answering the questionnaire (20€ in gift voucher).

Urban or rural status of the birthplace (parental address at time of birth) was assessed according to the population size of the municipality: a birthplace was considered urban when its municipality was composed of 2000 or more inhabitants, while the others were rural.

### 2.3 Study Population

Overall, 1367 participants and 640 mothers/relatives were enrolled in the study [18] (Figure S1). Among them, 44 participants did not meet inclusion criteria and were subsequently excluded: non GCNIS‐related TGCT (*N* = 21) and diagnosis other than a tumor (*N* = 4); *N* = 4 confirmed TGCT cases with time from diagnosis to inclusion > 12 months and *N* = 1 with missing diagnosis date; *N* = 1 group B control not born in mainland France; and *N* = 13 controls (8 group A and 5 group B) having reported personal history of cryptorchidism during interview. Among the 1323 eligible participants, 168 did not complete the telephone interview and were excluded: *N* = 48 TGCT cases, *N* = 46 group A controls and *N* = 74 group B controls. Reasons included refusal (*N* = 44), unreachable subjects after three telephone calls (*N* = 123) and 1 person who passed away prior to interview. Finally, 1124 participants and 31 additional mothers of participants not interviewed were included in the study population (*N* = 1155). In total, 472 TGCT cases, 393 group A and 290 group B controls were selected for the analysis.

### 2.4 Exposure Assessment

**FIGURE 1:**
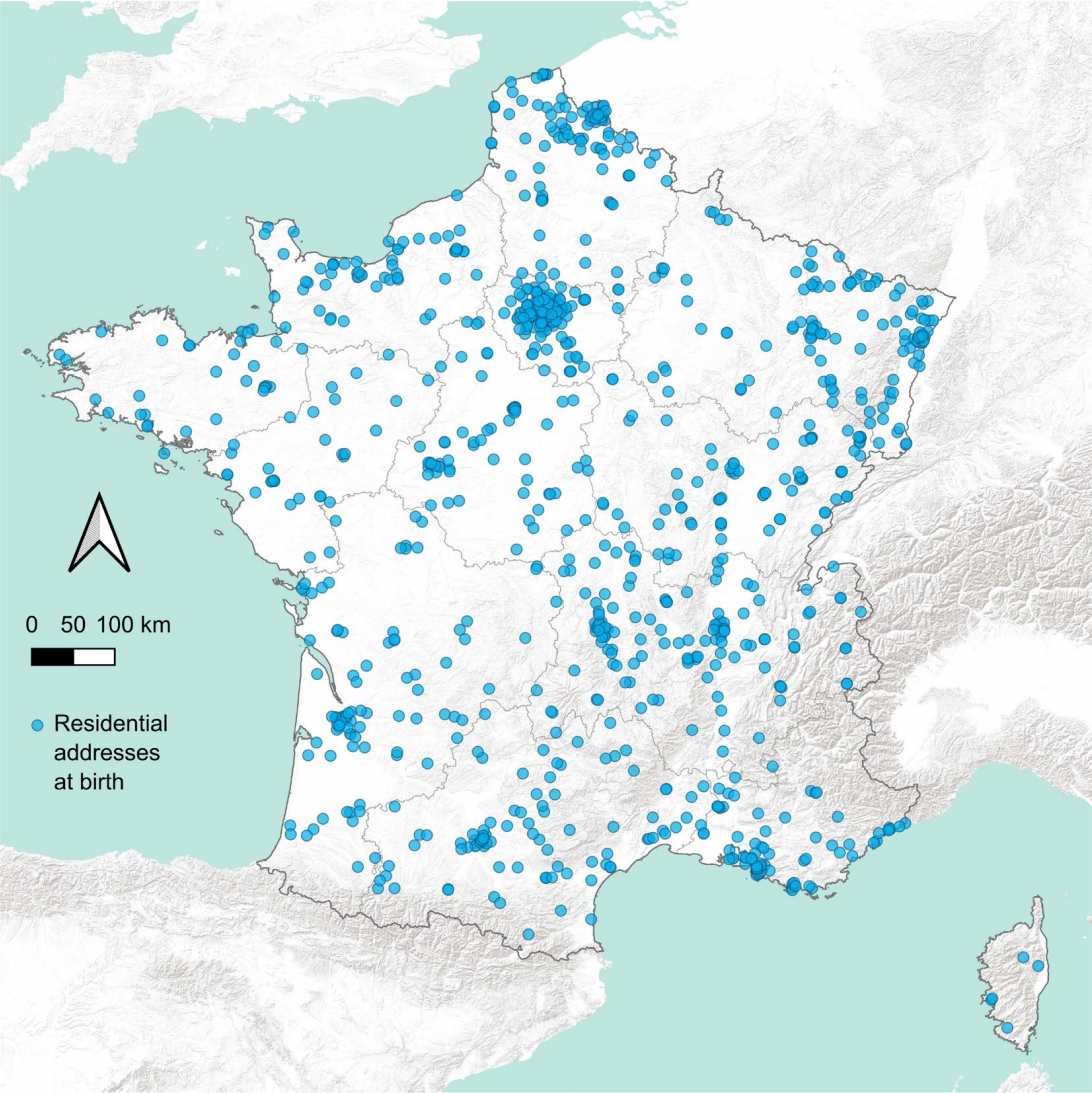
Distribution of TGCT cases and controls’ residential address at birth in mainland France, 1969–2000, *N* = 1155.

All residential addresses at birth declared by the participants (*N* = 1124) or their mothers (*N* = 31) were geocoded (X and Y coordinates, addresses) using ArcGIS software (ArcGIS Locator version 10.0, Environmental System Research Institute—ESRI, Redlands, CA, USA) and the address database BD Adresse for ArcGIS (v25.04) from the French National Institute of Geographic and Forest Information (IGN) [23] (Figure 1). Geocoding was performed by a trained technician blinded to the case–control status of the participants. Depending on the quality of the addresses collected, 53.4% of residence addresses were located at the exact address or the interpolated address and 20.6% at the street segment, while the remaining 26.0% of residence addresses had a lower level of positional accuracy (locality, administrative centre and postal code).

Two geographic determinants of exposure to agricultural pesticide drifts were integrated into the GIS: land use and crop acreage in 500 m and 1000 m buffers around residence at birth. In absence of a known distance threshold [24], 500 and 1000 m buffers have been found as relevant determinants of exposure and are two of the most used distances in the literature [25]. A semi‐automatic processing of historical black and white aerial images was developed through a software in order to determine land use at time of birth (from 1969 to 1999) of the study participants. The software development and functioning has been described in detail elsewhere [26, 27]. Briefly, in a first step, each image is segmented into patches, and an operator manually indicates examples of each land use category. Then, the software uses these examples to infer classification on the full image using texture features and supervised classification methods. The software can distinguish between the following land use categories: arable lands, orchards, vineyards, urban settings, waters, forests, and unknown (Figure S2). A total of 1297 photo‐aerial images for the birth year (±4 years) of the participants, covering a buffer zone of approximately 2 km around the birth address, were downloaded from the IGN Geoportail platform, which has been providing aerial photography online dating back to 1919 and georeferenced by specialized operators using the RGF93 coordinates system. Overall, 1111 (85.7%) images were entered into the software; 146 (11.3%) entirely consisted in urban settings or forest and were not analyzed, and 4 (0.3%) could not be treated due to poor image quality. Percentage of crop acreage was computed for arable lands, orchards and vineyards in 500 m and 1000 m buffers around residence at birth, and considered as a spatial proxy for exposure to agricultural pesticide drifts at birth. Presence (yes, i.e., percentage of land use > 0% /no) of arable lands, vineyards, and orchards was determined, as well as a global dichotomous exposure variable related to presence (yes/no) of the three types of crops. Additionally, tertiles of percentage of land use for the three types of crops and arable lands were computed, based on the distribution among controls; while number of subjects concerned were too low for orchards and vineyards to be categorized based on tertiles or median. Exposure was considered missing when more than 15% of the total buffer surface were lacking or when no image was available to cover the 500 m and 1000 m buffers (21 (1.8%) and 24 (2.1%) participants, respectively).

### 2.5 Statistical Analysis

Odds ratios (OR) and corresponding 95% confidence intervals (CI) for adult TGCT were estimated for presence of crops in a 500 m buffer around the residence at birth, overall and by type of crop (reference = absence of the crop, that is, other types of identified land use), using conditional logistic regression models. All models were conditioned for matching factors (hospital center administrative region (*N* = 11) and birth year grouped in 5‐year categories (*N* = 6)). We estimated OR and CI for TGCT overall and according to the histological TGCT subtype (seminoma and non‐seminoma). Heterogeneity of associations was tested using polytomous logistic regression for matched case–control studies (SAS macro *%subtype*), and *p*‐values were derived from the likelihood ratio test. For variables with less than five cases and five controls exposed, OR and CI were not estimated because of statistical uncertainty. Due to the low number of missing data for exposure variables (1.8%), these subjects were excluded from the analyses. All analyses were defined a priori. *p*‐values were two‐sided and the significance level was set at 0.05. SAS statistical software version 9.4 (SAS Institute Inc., Cary, NC, USA) was used for statistical analyses.

Covariates were identified from the literature [28] as possible early life risk factors for TGCT or confounding factors and considered for adjustment: maternal exposure to diethylstilbestrol during pregnancy (yes, no); maternal smoking during pregnancy (yes, no); breastfeeding (yes, no); birth weight (< 2500, 2500–3999, ≥ 4000 g); gestational age (≤ 36, > 36 weeks of pregnancy); birth order (first, second, third, fourth and more); sibship size (one, two, three, four and more brothers and sisters); multiple birth (yes, no); family history of testicular cancer (yes, no); family history of cryptorchidism (yes, no); and geographic origin (French by birth, by acquisition). In addition, broad categories of parental job titles (International Standard Classification of Occupations, ISCO‐1968 groups), industry sectors (*Nomenclature d’activités française*, NAF‐1999 sections) and social and occupational status (*Professions et categories socioprofessionnelles*, PCS‐2003 categories) at birth were tested. The urban/rural status of birthplace and the French geographic regions at birth were assessed as potential confounding factors because of the differing agricultural practices. Maternal domestic use of pesticides at the time of pregnancy and birth was also considered, although the covariate was available for 49.8% of the study population [18]. Age at diagnosis/inclusion was considered as a continuous variable. Each covariate was sequentially added to the model including the exposure variable (presence of crops in a 500 m buffer) and change in OR comparing models with and without the additional covariate was computed (Table S1). Covariates were retained if the change in OR was > 10%; as none of the covariates assessed met the criteria, results presented were computed from models not adjusted for those covariates.

Stratified analyses were performed according to: (i) birth cohorts (1970–1980, 1981–1990, and 1991–1999) to consider changes in agricultural practices and pesticide usages over time (these models were not conditioned for birth year); (ii) the season of first trimester of the mother’s pregnancy (start or end of first trimester in autumn/winter versus spring/summer, taking into consideration duration of the mother’s pregnancy) because spring and summer are spraying seasons [29]; (iii) the status of birthplace (urban, rural) because participants born in rural areas in the vicinity of agricultural activities may be exposed to higher levels of pesticides than participants born in urban areas [15]; and (iv) the geocoding accuracy (accurate (i.e., addresses geocoded at the exact address, the interpolated address, or at the street segment); less accurate (locality, administrative city, postal code and city)) to assess a potential misclassification bias of our exposure variables [23]. We assessed effect modification between exposure variables and each of the above mentioned strata variables using the likelihood ratio test comparing models with and without interaction terms.

To test the robustness of our results, models were run in different subgroups: (i) excluding TGCT cases with personal history of cryptorchidism (*N* = 40); (ii) excluding cases and controls having reported family history of testicular cancer (*N* = 49) and/or cryptorchidism (*N* = 46); and (iii) excluding TGCT cases not confirmed by pathology reports (*N* = 43). Finally, we investigated the effect of the presence of crops in a 1000 m buffer around residence at birth on adult TGCT risk.

## 3 Results

Characteristics of TGCT cases and control groups A and B are described in Table 1 and Table S2. Low birthweight (< 2500 g) was reported for 5.3% of cases, and for 4.5% and 5.1% of Group A and Group B controls, respectively. The participants were mostly first born (from 42.7% to 47.2%) and more cases than controls were born from a multiple pregnancy (4.4% vs. 2.0%/3.1%). Family histories of TGCT and cryptorchidism were more frequent among cases than among Group A and Group B controls (7.0% vs. 1.8%/3.1%, and 5.9% vs. 2.5%/2.8%, respectively). There was no difference between TGCT cases and controls in parental occupations and in birthplace characteristics. Groups A and B controls only differed in parental social and occupational categories, with more workers among Group A than Group B controls (11.7% vs. 3.4% in mothers; 41.0% vs. 25.5% in fathers). There were more cases than controls aged below 25 years (14.8% vs. 5.6%/4.5%) and less cases than controls aged 31 to 35 years (24.8% vs. 34.1%/37.6%) (Table 2). Among TGCT cases, 43.0% were non‐seminomas with a peak age at diagnosis at 26–30 years, and 47.7% were seminomas with a peak age at diagnosis of 31–35 years.

**TABLE 1:** Characteristics of TGCT cases and controls (Group A and Group B), *N* = 1155, case–control study, France, 2015–2018.

	Group A controls (*N* = 393)	Group B controls (*N* = 290)	TGCT cases (*N* = 472)
	*n* (%)	*n* (%)	*n* (%)
Prenatal characteristics			
Maternal use of diethylstilbestrol during pregnancy^a^			
No	142 (36.1)	129 (44.5)	296 (62.7)
Yes	0 (0.0)	0 (0.0)	1 (0.2)
Missing	251 (63.9)	161 (55.5)	175 (37.1)
Maternal smoking during pregnancy^a^			
No	122 (31.0)	110 (37.9)	252 (53.4)
Yes	23 (5.9)	19 (6.6)	52 (11.0)
Missing	248 (63.1)	161 (55.5)	168 (35.6)
Maternal age (years), mean (SD)^a^	27 (4.7)	27.8 (4.6)	27.6 (4.7)
Missing	248 (63.1)	161 (55.5)	168 (35.6)
Paternal age (years), mean (SD)^a^	29.5 (5.3)	30.2 (5.4)	29.8 (4.8)
Missing	313 (79.6)	228 (78.6)	337 (71.4)
Perinatal characteristics			
Inguinal hernia			
No	362 (92.1)	275 (94.8)	414 (87.7)
Yes	22 (5.6)	10 (3.4)	39 (8.3)
Missing	9 (2.3)	5 (1.7)	19 (4.0)
Birth weight			
< 2500 g	20 (5.1)	13 (4.5)	25 (5.3)
[2500–4000] g	318 (80.9)	243 (83.8)	356 (75.4)
≥ 4000 g	32 (8.1)	26 (9.0)	46 (9.7)
Missing	23 (5.9)	8 (2.8)	45 (9.5)
Gestational age			
≤ 36 weeks	23 (5.9)	11 (3.8)	32 (6.8)
> 36 weeks	358 (91.1)	271 (93.4)	415 (87.9)
Missing	12 (3.1)	8 (2.8)	25 (5.3)
Birth order			
First	168 (42.7)	137 (47.2)	213 (45.1)
Second	135 (34.4)	89 (30.7)	163 (34.5)
Third	51 (13.0)	42 (14.5)	63 (13.3)
Fourth and more	30 (7.6)	18 (6.2)	15 (3.2)
Missing	9 (2.3)	4 (1.4)	18 (3.8)
Sibship size			
1	36 (9.2)	23 (7.9)	25 (5.3)
2	139 (35.4)	115 (39.7)	192 (40.7)
3	116 (29.5)	90 (31.0)	153 (32.4)
≥ 4	92 (23.4)	58 (20.0)	84 (17.8)
Missing	10 (2.5)	4 (1.4)	18 (3.8)
Birth from multiple pregnancy			
No	375 (95.4)	277 (95.5)	433 (91.7)
Yes	8 (2.0)	9 (3.1)	21 (4.4)
Missing	10 (2.5)	4 (1.4)	18 (3.8)
Birth cohort			
1970–1980	138 (35.1)	87 (30.0)	139 (29.4)
1981–1990	220 (56.0)	182 (62.8)	256 (54.2)
1991–2000	35 (8.9)	21 (7.2)	77 (16.3)
Predisposing characteristics			
Geographic origin			
French by birth	380 (96.7)	281 (96.9)	449 (95.1)
French by acquisition	4 (1.0)	5 (1.7)	5 (1.1)
Missing	9 (2.3)	4 (1.4)	18 (3.8)
Family history of TGCT			
No	375 (95.4)	276 (95.2)	419 (88.8)
Yes	7 (1.8)	9 (3.1)	33 (7.0)
Missing	11 (2.8)	5 (1.7)	20 (4.2)
Family history of cryptorchidism			
No	369 (93.9)	276 (95.2)	422 (89.4)
Yes	10 (2.5)	8 (2.8)	28 (5.9)
Missing	14 (3.6)	6 (2.1)	22 (4.7)
Residential characteristics			
Urban status of the place of birth, commune level			
No	99 (25.2)	58 (20.0)	120 (25.4)
Yes	294 (74.8)	232 (80.0)	352 (74.6)
Geocoding accuracy of residence at birth			
Address	191 (48.6)	154 (53.1)	236 (50.0)
Interpolated address	14 (3.6)	12 (4.1)	10 (2.1)
Street segment	83 (21.1)	48 (16.6)	107 (22.7)
Locality	32 (8.1)	17 (5.9)	37 (7.8)
Administrative city	69 (17.6)	57 (19.7)	79 (16.7)
Postal code	4 (1.0)	1 (0.3)	2 (0.4)
City	0 (0.0)	1 (0.3)	1 (0.2)

^a^Missing data are due to the absence of mothers’ interview (*N* = 577).

**TABLE 2:** Age distribution (years) of TGCT cases, overall and by subtypes, and Group A and Group B controls at diagnosis/inclusion, case–control study, *N* = 1155, France, 2015–2018.

Age at diagnosis/inclusion (years)	Group A controls	Group B controls	TGCT cases^a^	Non‐seminomas	Seminomas
	*n* (%)	*n* (%)	*n* (%)	*n* (%)	*n* (%)
≤ 25	22 (5.6)	13 (4.5)	70 (14.8)	52 (25.6)	18 (8.0)
26–30	83 (21.1)	66 (22.8)	113 (23.9)	61 (30.0)	52 (23.1)
31–35	134 (34.1)	109 (37.6)	117 (24.8)	48 (23.6)	68 (30.2)
36–40	96 (24.4)	67 (23.1)	86 (18.2)	29 (14.3)	57 (25.3)
≥ 41	58 (14.8)	35 (12.1)	43 (9.1)	13 (6.4)	30 (13.3)
Missing	0 (0.0)	0 (0.0)	43 (9.1)	0 (0.0)	0 (0.0)
Total	393 (100.0)	290 (100.0)	472 (100.0)	203 (100.0)	225 (100.0)

^a^ Include *N* = 1 confirmed TGCT case of regressed germ cell tumors (GCNIS‐related TGCT, unknown subtype) and *N* = 43 TGCT cases not confirmed by pathology reports.

The median (Q1–Q3) of percentage of area of arable land in a 500 m buffer around residence at birth was 2.8% (0.0–19.2), 2.9% (0.0–17.5) and 2.7% (0.0–18.7) among TGCT cases, Group A and Group B controls (Table 3), while the median (Q1–Q3) was 0.0% among cases and controls for orchards and vineyards.

**TABLE 3:** Distribution of land use around residence at birth by case–control status, *N* = 1155, France, 2015–2018.

Exposure	Group A controls (*N* = 393)	Group B controls (*N* = 290)	TGCT cases (*N* = 472)
500 m buffer around residence at birth			
Area of arable land (%)			
Min	0.0	0.0	0.0
Max	84.8	85.4	98.5
Q1	0.0	0.0	0.0
Median	2.9	2.7	2.8
Q3	17.5	18.7	19.2
Area of orchards (%)			
Min	0.0	0.0	0.0
Max	33.5	8.3	52.2
Q1	0.0	0.0	0.0
Median	0.0	0.0	0.0
Q3	0.0	0.0	0.0
Area of vineyards (%)			
Min	0.0	0.0	0.0
Max	38.2	40.0	66.3
Q1	0.0	0.0	0.0
Median	0.0	0.0	0.0
Q3	0.0	0.0	0.0
1000 m buffer around residence at birth			
Area of arable land (%)			
Min	0.0	0.0	0.0
Max	83.0	91.5	96.7
Q1	0.0	0.0	0.1
Median	6.5	5.2	6.2
Q3	25.5	30.5	27.3
Area of orchards (%)			
Min	0.0	0.0	0.0
Max	37.9	13.9	38.2
Q1	0.0	0.0	0.0
Median	0.0	0.0	0.0
Q3	0.0	0.0	0.0
Area of vineyards (%)			
Min	0.0	0.0	0.0
Max	46.3	43.0	61.5
Q1	0.0	0.0	0.0
Median	0.0	0.0	0.0
Q3	0.0	0.0	0.0

Overall, 71.3% of cases and controls had agricultural lands in the buffer around residence at birth (70.0% had arable lands, 9.2% vineyards and 8.7% orchards) (Table 4). There was no association between TGCT risk and presence of arable lands (OR = 1.14, CI = 0.86–1.52), vineyards (OR = 0.88, CI = 0.56–1.36), as well as agricultural surfaces overall (OR = 1.13, CI = 0.84–1.51), but a borderline significant association with orchards was found (OR = 1.51, CI = 0.99–2.32). We further observed a statistically significant increase in seminoma risk for presence of orchards (OR = 1.78, CI = 1.07–2.97), and the association that was found for non‐seminoma for orchards was slightly weaker (OR = 1.46, CI = 0.79–2.69), but there was no heterogeneity between the two histological subtypes (*p*‐het = 0.39). Neither seminoma or non‐seminoma risks were associated with the presence of arable lands, vineyards, or agricultural surfaces overall.

**TABLE 4:** Odds ratios and 95% confidence intervals for TGCT associated with GIS‐based land use in a 500 m buffer around place of residence at birth, overall and according to histological subtypes, case–control study, *N* = 1155, France, 2015–2018.

	Total (*N* = 1155)	Group A controls (*N* = 393)	Group B controls (*N* = 290)	All TGCT cases (*N* = 472)			Non‐seminomas (*N* = 203)			Seminomas (*N* = 225)			*p*‐HET^b^
	*n* (%)	*n* (%)	*n* (%)	*n* (%)	OR^a^	(95% CI)	*n* (%)	OR^a^	(95% CI)	*n* (%)	OR^a^	(95% CI)	
Presence of arable lands, orchards and vineyards													0.55
No	311 (26.9)	103 (26.2)	90 (31)	118 (25.0)	1.00		54 (26.6)	1.00		52 (23.1)	1.00		
Yes	823 (71.3)	284 (72.3)	196 (67.6)	343 (72.7)	1.13	(0.84, 1.51)	145 (71.4)	1.03	(0.69, 1.54)	167 (74.2)	1.22	(0.83, 1.78)	
Missing	21 (1.8)	6 (1.5)	4 (1.4)	11 (2.3)	—	—	4 (2.0)	—	—	6 (2.7)	—	—	
Presence of arable lands													0.42
No	326 (28.2)	110 (28.0)	92 (31.7)	124 (26.3)	1.00		57 (28.1)	1.00		54 (24.0)	1.00		
Yes	808 (70.0)	277 (70.5)	194 (66.9)	337 (71.4)	1.14	(0.86, 1.52)	142 (70.0)	1.02	(0.68, 1.52)	165 (73.3)	1.28	(0.88, 1.86)	
Missing	21 (1.8)	6 (1.5)	4 (1.4)	11 (2.3)	—	—	4 (2.0)	—	—	6 (2.7)	—	—	
Presence of orchards													0.62
No	1034 (89.5)	353 (89.8)	269 (92.8)	412 (87.3)	1.00		180 (88.7)	1.00		191 (84.9)	1.00		
Yes	100 (8.7)	34 (8.7)	17 (5.9)	49 (10.4)	1.51	(0.99, 2.32)	19 (9.4)	1.46	(0.79, 2.69)	28 (12.4)	1.78	(1.07, 2.97)	
Missing	21 (1.8)	6 (1.5)	4 (1.4)	11 (2.3)	—	—	4 (2.0)	—	—	6 (2.7)	—	—	
Presence of vineyards													0.39
No	1028 (89.0)	340 (86.5)	269 (92.8)	419 (88.8)	1.00		184 (90.6)	1.00		195 (86.7)	1.00		
Yes	106 (9.2)	47 (12.0)	17 (5.9)	42 (8.9)	0.88	(0.56, 1.36)	15 (7.4)	0.72	(0.38, 1.38)	24 (10.7)	1.04	(0.61, 1.79)	
Missing	21 (1.8)	6 (1.5)	4 (1.4)	11 (2.3)	—	—	4 (2.0)	—	—	6 (2.7)	—	—	
Area of arable land occupation, tertiles (%)													0.78
[0.00–0.12]	352 (30.5)	119 (30.3)	97 (33.4)	136 (28.8)	1.00		61 (30.0)	1.00		60 (26.7)	1.00		
[0.12–11.44]	400 (34.6)	136 (34.6)	92 (31.7)	172 (36.4)	1.19	(0.87, 1.62)	72 (35.5)	1.07	(0.70, 1.66)	84 (37.3)	1.29	(0.86, 1.94)	
> 11.44	382 (33.1)	132 (33.6)	97 (33.4)	153 (32.4)	1.03	(0.75, 1.41)	66 (32.5)	0.96	(0.62, 1.50)	75 (33.3)	1.17	(0.77, 1.77)	
Missing	21 (1.8)	6 (1.5)	4 (1.4)	11 (2.3)	—	—	4 (2.0)	—	—	6 (2.7)	—	—	
Area of land occupation, tertiles (%)													0.57
[0.00–0.30]	363 (31.4)	125 (31.8)	99 (34.1)	139 (29.4)	1.00		63 (31.0)	1.00		61 (27.1)	1.00		
[0.30–13.66]	387 (33.5)	133 (33.8)	91 (31.4)	163 (34.5)	1.14	(0.84, 1.56)	65 (32.0)	0.96	(0.62, 1.48)	83 (36.9)	1.32	(0.88, 1.97)	
> 13.66	384 (33.2)	129 (32.8)	96 (33.1)	159 (33.7)	1.09	(0.80, 1.49)	71 (35.0)	1.05	(0.68, 1.62)	75 (33.3)	1.20	(0.79, 1.80)	
Missing	21 (1.8)	6 (1.5)	4 (1.4)	11 (2.3)	—	—	4 (2.0)	—	—	6 (2.7)	—	—	

^a^ Estimates obtained comparing TGCT cases to Group A and Group B controls combined. Analysis was restricted to subjects with no missing data for the exposure variable (1.8% excluded).

^b^ *p*‐value for heterogeneity derived from the Likelihood Ratio Test, comparing seminoma versus non‐seminoma tumors.

There was no effect modification by birth cohorts and no association was observed overall, except for an increased OR for the presence of orchards among participants born between 1981 and 1990 (OR = 2.02, CI = 1.16–3.52, *p*‐int = 0.32) (Table S3). We found no effect modification by season of pregnancy first trimester, except for presence of orchards (*p*‐int = 0.03) with an increased OR among participants for whom part of the first trimester of fetal development occurred in spring or summer (OR = 1.70, CI = 1.03–2.82) (Table S4). When stratifying on the rural or urban status of birthplace, no effect modification nor association was observed for presence of arable lands, orchards and vineyards (Table S5). The stratified analysis on geocoding accuracy did not show any effect modification nor association (Table S6).

Similar results were observed in sensitivity analyses. In the subgroup restricted to cases and controls with no personal history of cryptorchidism, no association was found overall except for a statistically significant increased OR for presence of orchards (OR = 1.57, CI = 1.01–2.44) (Table S7). The same association was observed in the subgroup with no family history of cryptorchidism and testicular cancer for presence of orchards (OR = 1.59, CI = 1.02–2.48). Excluding TGCT cases not confirmed by pathology reports did not modify our results, and the positive association with presence of orchards was statistically significant with an OR = 1.61 (CI = 1.05–2.49). Table S8 presents the ORs for agricultural surfaces in 1000 m buffer around residence at birth. There was no association with TGCT risk, and the association previously observed for seminoma with presence of orchards in a 500 m buffer was substantially weaker using a 1000 m buffer (OR = 1.33, CI = 0.83–2.12, *p*‐het = 0.95).

## 4 Discussion

Our study found no overall association between presence of agricultural crops in a 500 m buffer around the residence at birth and the risk of adult TGCT. An increase in risk was suggested for presence of orchards, which was slightly stronger for seminomas than for non‐seminomas and confirmed in subgroup and sensitivity analyses. Additionally, an increase in TGCT risk for presence of orchards was suggested for subjects with first trimester in utero development during spring and summer. Our findings were not sensitive to accuracy of geocoding of birth addresses.

Evidence from the literature is limited regarding the effect of early pesticide exposure on TGCT risk [30]. However, recent publications have reported elevated risk of TGCT associated with residential pesticide exposure: a meta‐analysis showed increased risks of testicular cancer and seminoma and non‐seminoma subtypes in male offspring associated with maternal exposure to grouped endocrine disrupting chemicals, in particular organochlorines [13]. Major limitations to the studies included in the meta‐analysis were risk of bias and no consideration of mixed exposures. In a registry‐based case–control study in California US, positive associations for TGCT in 15–19 years old adolescents were reported with prenatal quantities of agricultural insecticides acephate, malathion and carbaryl, applied in 1 and 3 km buffers around residence at birth [14]. A population‐based case control study conducted in several regions of Andalusia Spain, reported that populations living in areas of intensive agricultural activities in greenhouses, with elevated use of pesticides, were at higher risk of developing testicular cancer than those living in low exposure areas [31]. Regarding other testicular dysgenesis related outcomes, an increased risk of hypospadias was found in children whose mothers lived close to vineyards during pregnancy [32], while a modest increase in risk of cryptorchidism was observed for prenatal residential proximity to orchards [33].

Our results showed a stronger association for presence of orchards in the subgroup of men whose mothers’ first trimester of pregnancy (or part of) occurred during the spraying season (spring and summer). This may be explained by the higher levels of pesticide exposure reported during the spraying season compared to the non‐spraying season [15, 34], and the disruption of the masculinization programming window by gestational exposure to chemicals with anti‐androgenic properties such as pesticides, which have been shown to induce testicular dysgenesis in experimental studies [9, 35].

Our main analysis was performed assuming that the pesticides applied were the same whatever the birth period, which was likely not true based on sales data and the literature. Indeed, we observed an increased risk of TGCT for presence of orchards in the 1981–1990 birth cohort, which could be due to changes in products, in the quantity of pesticides, or both. Our results are in line with the changes in use over time: in France, as well as in the United States, the use of pesticides has increased continually since the 1960’s, reached a peak in the 1980’s and started to decrease in the 1990’s [16, 36]. However, there was no statistically significant effect modification by birth cohorts; the 1981–1990 period was also the largest one in terms of number of participants, whereas the number of cases and controls may have been too low in the last period (1991–1999), resulting in limited statistical power.

Agricultural land use was highly prevalent in the study population, representing 71.3% of both cases and controls within 500‐m buffers, largely driven by arable lands (70.0%), and remained stable across birth cohorts. Presence of orchards and vineyards was much lower (8.7% and 9.2%). Similarly, the French study by Cognez et al. reported that 83% of mothers were exposed to at least one crop likely to be treated by pesticides during their pregnancy; although the buffer size was 1000 m and the period of pregnancies was more recent [33]. These numbers are also consistent with the French agricultural censuses which reported an average density of area used for agriculture in municipalities of about 60% in 1970 and 55% in 2000.

Grapes and vines were the most intensive sector of pesticide use in France, with 44 to 33 kg/ha of pesticides quantity used between 1995 and 2003, followed by arboriculture (from 28 to 16 kg/ha) and arable (from 2 to 2.5 kg/ha) and vegetable lands (from 8 to 7 kg/ha) [37]. The yearly average number of pesticide treatments in orchards ranges from 4 in citrus to 40 in apples. The surface receiving at least one pesticide treatment is also very high, from 84.0% for citrus to 99.9% for plums [11]. In the 1990s, while the most important active substance used was inorganic sulfur fungicide in vineyards and the herbicides bentazone and pendimethalin in arable crops, orchards were mostly sprayed with fungicides: sulfur, mancozeb (group: carbamates), captan (group: phthalimides), and iprodione (group: dicarboxymids) [37].

Mancozeb is an endocrine disrupting fungicide that has been shown to decrease sperm motility and count, inhibit sex steroid synthesis through inhibition of cytochrome P450 leading to the downregulation of androgen and estrogen receptor signaling and induce oxidative damage and apoptosis in testes in animal and in vitro studies [38, 39]. Similar adverse effects on male reproductive functions have been observed after iprodione exposure in rats, but with limited evidence [40]. Although some studies have found that fungicide captan ingestion may lead to inhibition of DNA synthesis in testes and severe testicular atrophy in animal studies [41], there is very little literature on the health effects of captan exposure. Sulfur is a common pesticide widely applied in agriculture and is known to be a relatively harmless substance because of its low toxicity to humans and wildlife. Due to its safety record and as a natural mineral element, sulfur has also been used in organic farming [42]. There is no evidence that sulfur might contribute to TGCT etiology.

Recent reviews and studies reported consistent associations between individual exposure to certain pesticides and diminished sperm parameters (concentration, motility, morphology and DNA integrity), disrupted reproductive hormones levels and reduced male fertility in epidemiological studies [43, 44]. In particular, the inhibition of androgens’ action by anti‐androgenic pesticides during fetal masculinization programming window may lead to dysfunction of Sertoli and Leyding cells and may explain testicular dysgenesis later in life [9]. Recently, maternal exposure to endocrine disruptors has been hypothesized to predispose to the risk of developing testicular cancer [43]. Current scientific evidence in humans is limited and further investigation is required on the effects of endocrine disrupting chemicals and pesticides on the onset of testicular dysgenesis and testicular cancer [45].

Our study is limited by the lack of historical data on specific pesticide chemicals (type and active ingredient) as well as the quantity of pesticides applied on the lands and the number of annual treatments. We were able to define the type of lands; however, it was not possible to specifically identify which crop had grown on it, in particular in arable lands and orchards. While we were unable to differentiate conventional farming (which uses synthetic chemicals) from organic agriculture (which uses mineral fungicides and plant‐based insecticides or of microbiological origins), the latter was marginal for the birth years of the study participants, covering 0.42% and 1.4% of the French utilized agricultural area in 1995 and 2001, respectively, and even less before the 90s [46]. Moreover, some of the biocontrol products approved for agricultural farming in France have been shown to have endocrine disrupting effects [47]. The use of our indirect indicators for agricultural pesticides exposure may have led to non‐differential misclassification bias leading to a possible attenuation of association, if there was one [48]. While our main analyses were based on pre‐specified hypotheses, multiple testing may have elevated the risk of type I error, meaning that some of the observed associations could have arisen by chance. However, the various land‐use variables were correlated and should not be considered entirely independent.

Another limitation concerns the use of GIS in epidemiological studies, and in particular the geocoding inaccuracy—due to lower quality of the addresses collected—that can also cause exposure misclassification [25]. To address this limitation, we stratified our analysis on geocoding accuracy (accurate vs. less accurate), and results did not change. Meteorological data (wind direction, wind velocity) and topographic data (barriers) may be determinants that can influence quality and accuracy of GIS exposure assessment [25]. We tested additional parameters including wind direction, and because they did not modify exposure level ranking, they were not integrated into the GIS [26]. Comparison of the land use determined using our software to two other databases (Corine Land Cover from 1990 and OCS Picardie from 1992) showed high agreement for arable lands, but rather low agreement for vineyards and orchards, probably due to the low resolution in Corine Land Cover (25 ha) that prevented the identification of small parcels, frequent in arboriculture and viticulture [26]. To improve the accuracy of pesticide exposure assessment, future studies could integrate high‐resolution land use data, detailed application logs and modeled drift estimates generated by validated atmospheric models (such as agDRFIT or AGDISP). Although our study did not implement this layered approach due to a lack of data for the year of birth for most participants, these models were specifically designed to assess downwind deposition and off‐target drift from aerial, ground boom, and orchard/vineyard airblast applications [49].

Recruitment of participants was challenging in our study, mostly because testicular cancer and fertility are sensitive topics. Our pilot study showed that recruiting cases and controls from French university hospitals was the most promising approach to increase consent and minimize nonresponse bias [50]. Yet, this approach may yield a sampling frame that does not fully represent the source population from which the cases originate. Although fecund controls were selected to reduce the risk of recruiting individuals with testicular dysgenesis‐related disorders, it may have introduced selection bias potentially leading to under‐ or overestimation of the observed associations and limiting the generalizability to the source population of TGCT cases. Moreover, by conditioning on the hospital centers’ administrative region, we attempted to avoid over‐matching of cases and controls. However, within‐region heterogeneity in exposures could result in residual confounding if relevant environmental or socioeconomic factors vary at a finer spatial scale.

To our knowledge, this is the largest study conducted on TGCT covering the whole mainland French territory. It benefits from the prospective and multi‐centric recruitment of cases and controls, the analysis of histological subtypes seminoma and non‐seminoma and the assessment of confounding from a large data collection. Other sources of pesticides exposures, including parental occupation and domestic use [18], were tested and did not change risk estimates. In this study, retrospective exposure assessment was a challenge. However, even in the absence of historical data, we were able to reconstruct past land use from 1969 onward through the development of a specific software [27]. We used GIS methods to determine exposure at an individual level and geocoding accuracy was assessed to limit misclassification bias [23].

In conclusion, our study reported no overall association between TGCT and the presence of agricultural crops around residence at birth. However, we observed an increased risk of TGCT, particularly seminomas, associated with the presence of orchards within 500 m of the residence at birth. This association was especially marked among subjects whose conception or first trimester occurred during spring–summer, a period corresponding to peak pesticide application. These findings reinforce the importance of existing measures to minimize drift and runoff, and support stricter buffer zones between treated fields and residential or school areas. They also call for seasonal restrictions on pesticide uses near sensitive areas during peak periods, alongside public awareness campaigns. Further large‐scale studies, pooling existing datasets, are required to clarify the role of specific exposure windows, pesticide active ingredients and tumor subtypes. Finally, access to detailed historical pesticide application records, finer resolution of crop types, and metrics that capture pesticide mixtures would improve risk assessment and guide more effective, evidence‐based prevention strategies.

## Author Contributions

**Aurélie M. N. Danjou:** formal analysis, data curation, writing – original draft, writing – review and editing, visualization, project administration. **Astrid Coste:** writing – review and editing, project administration. **Lény Grassot:** resources, writing – review and editing. **Elodie Faure:** resources, writing – review and editing. **Carlos Crispim‐Junior:** resources, writing – review and editing. **Laure Tougne Rodet:** resources, writing – review and editing. **Olivia Pérol:** conceptualization, methodology, writing – review and editing. **Rémi Béranger:** conceptualization, methodology, writing – review and editing. **Jeanne Perrin:** writing – review and editing, resources. **Barbara Charbotel:** conceptualization, writing – review and editing. **Joachim Schüz:** conceptualization, methodology, writing – review and editing, funding acquisition, supervision, project administration. **Béatrice Fervers:** conceptualization, methodology, writing – review and editing, supervision, funding acquisition, project administration. TESTIS Study group: Resources. All authors have read the manuscript, agree the work is ready for submission to a journal and accept responsibility for the manuscript’s contents.

## Funding

The project was supported by funding from the Institut National Du Cancer/French National Cancer Institute (INCa, No. 2013‐143) and the Institut National de la Santé et de la Recherche Médicale (Inserm, No. ENV201306/CLB). The development of the method for exposure assessment was funded by the LABEX IMU (ANR‐10‐LABX‐0088) of Université de Lyon, within the Investissements d’Avenir program (ANR‐11‐IDEX‐0007) and the French Ligue Contre le Cancer (AAPPVN2021.LCC/BF).

## Disclosure

Where authors are identified as personnel of the International Agency for Research on Cancer/World Health Organization, the authors alone are responsible for the views expressed in this article and they do not necessarily represent the decisions, policy or views of the International Agency for Research on Cancer/World Health Organization.

## Ethics Statement

The study received ethical approval from the French Ethics Committee (ref. no. A14‐94), the French national agency for medicines and health products safety (ref. no. 140184B‐12), and the IARC Ethics Committee (ref. no. 14‐26) and was declared to the Commission nationale de l’Informatique et des Libertés (MR‐001, ref. no. 2016‐177), as well as registered at http://www.clinicaltrials.gov (NCT02109926).

## Consent

Cases and controls were asked for their permission to contact their mother or, when the mother was not available, the closest relative (in family terms) to participate in the study. All participants and mothers/relatives included in the analyses provided written informed consent.

## Conflicts of Interest

The authors declare no conflicts of interest.

## Supporting information


**Table S1:** Odds ratios (OR) and 95% confidence intervals (CI) for GCNIS‐related TGCT associated with presence of arable lands, orchards and vineyards in a 500 m buffer around residence at birth, change in estimates, case–control study, *N* = 1155, France, 2015–2018.
**Table S2:** Continued from Table 1.
**Table S3:** Odds ratios (OR) and 95% confidence intervals (CI) for GCNIS‐related TGCT associated with GIS‐based land use in a 500 m buffer around place of residence at birth, stratified analysis on birth cohort, case–control study, *N* = 1155 France, 2015–2018.
**Table S4:** Odds ratios (OR) and 95% confidence intervals (CI) for GCNIS‐related TGCT associated with GIS‐based land use in a 500 m buffer around place of residence at birth, stratified analyses on season of pregnancy, case–control study, *N* = 1155, France, 2015–2018.
**Table S5:** Odds ratios (OR) and 95% confidence intervals (CI) for GCNIS‐related TGCT associated with GIS‐based land use in a 500 m buffer around place of residence at birth, stratified analyses on birthplace status, case–control study, *N* = 1155, France, 2015–2018.
**Table S6:** Odds ratios (OR) and 95% confidence intervals (CI) for GCNIS‐related TGCT associated with GIS‐based land use in a 500 m buffer around place of residence at birth, stratified analyses on geocoding accuracy, case–control study, *N* = 1155, France, 2015–2018.
**Table S7:** Odds ratios (OR) and 95% confidence intervals (CI) for GCNIS‐related TGCT associated with GIS‐based land use in a 500 m buffer around place of residence at birth, sensitivity analyses, case–control study, France, 2015–2018.
**Table S8:** Odds ratios (OR) and 95% confidence intervals (CI) for GCNIS‐related TGCT associated with GIS‐based land use in a 1000 m buffer around place of residence at birth, sensitivity analyses, case–control study, *N* = 1155, France, 2015–2018.
**Figure S1:** Flowchart of the selection of the study population, TESTIS, France, 2015–2018.
**Figure S2:** Illustration of the classification of land‐use using the GOURAMIC software in buffers around address of residence, TESTIS, France.

## Data Availability

The datasets used and/or analyzed during the current study are available from the corresponding author on reasonable request.
