# Supplementary material for: Residence Near Agricultural Crops at Birth and Risk of Adult Testicular Germ Cell Tumors: A French Nationwide Case–Control Study Using Historical Aerial GIS Data
Source: Int J Cancer. 2026 May 18;159(5):1153–66. doi: 10.1002/ijc.70469 (PMC13340941; doi:10.1002/ijc.70469)

Residence near agricultural crops at birth and risk of adult germ cell tumours : a French nationwide case-control study using historical aerial GIS data

International Journal of Cancer

Authors:
Aurélie MN DANJOU, Astrid COSTE, Lény GRASSOT, Elodie FAURE, Carlos CRISPIM-JUNIOR, Laure TOUGNE RODET, Olivia PEROL, Rémi BERANGER, Jeanne PERRIN, Barbara CHARBOTEL, Joachim SCHUZ, Béatrice FERVERS, and the TESTIS Study Group

Table of Contents:

Supplementary tables:
- Table S1. Odds ratios (OR) and 95% confidence intervals (CI) for GCNIS-related TGCT associated with presence of arable lands, orchards and vineyards in a 500 m buffer around residence at birth, change in estimates, case-control study, N=1155, France, 2015-2018
- Table S2. Continued from Table 1
- Table S3. Odds ratios (OR) and 95% confidence intervals (CI) for GCNIS-related TGCT associated with GIS-based land use in a 500 m buffer around place of residence at birth, stratified analysis on birth cohort, case-control study, N=1155 France, 2015-2018
- Table S4. Odds ratios (OR) and 95% confidence intervals (CI) for GCNIS-related TGCT associated with GIS-based land use in a 500 m buffer around place of residence at birth, stratified analyses on season of pregnancy, case-control study, N=1155, France, 2015-2018
- Table S5. Odds ratios (OR) and 95% confidence intervals (CI) for GCNIS-related TGCT associated with GIS-based land use in a 500 m buffer around place of residence at birth, stratified analyses on birthplace status, case-control study, N=1155, France, 2015-2018
- Table S6. Odds ratios (OR) and 95% confidence intervals (CI) for GCNIS-related TGCT associated with GIS-based land use in a 500 m buffer around place of residence at birth, stratified analyses on geocoding accuracy, case-control study, N=1155, France, 2015-2018
- Table S7. Odds ratios (OR) and 95% confidence intervals (CI) for GCNIS-related TGCT associated with GIS-based land use in a 500 m buffer around place of residence at birth, sensitivity analyses, case-control study, France, 2015-2018
- Table S8. Odds ratios (OR) and 95% confidence intervals (CI) for GCNIS-related TGCT associated with GIS-based land use in a 1000 m buffer around place of residence at birth, sensitivity analyses, case-control study, N=1155, France, 2015-2018

Supplementary figures:
- Figure S1. Flowchart of the selection of the study population, TESTIS, France, 2015-2018.
- Figure S2. Illustration of the classification of land-use using the GOURAMIC® software in buffers around address of residence, TESTIS, France.

Table S1. Odds ratios (OR) and 95% confidence intervals (CI) for GCNIS-related TGCT associated with presence of arable lands, orchards and vineyards in a 500 m buffer around residence at birth, change in estimates, case-control study, N=1155, France, 2015-2018

	**Presence of arable lands, orchards and vineyards**		
	OR[a]	Lower 95% CI	Upper 95% CI
Univariate model	1.13	0.84	1.51
**Adjustment for**			
Maternal use of diethylstilbestrol during pregnancy	1.13	0.84	1.52
Maternal smoking during during pregnancy	1.12	0.83	1.52
Maternal age	1.12	0.83	1.52
Paternal age	1.17	0.87	1.56
Inguinal hernia	1.14	0.85	1.54
Hypospadia	1.12	0.83	1.50
Birth weight	1.14	0.85	1.53
Gestational age	1.10	0.81	1.48
Birth order	1.12	0.83	1.51
Sibship size	1.08	0.81	1.46
Birth from multiple pregnancy	1.11	0.83	1.50
Geographic origin	1.12	0.83	1.50
Family history of TGCT	1.15	0.85	1.56
Family history of cryptorchidism	1.08	0.81	1.46
Maternal job at birth (ISCO-1968 codes)	1.16	0.86	1.55
Paternal job at birth (ISCO-1968 codes)	1.15	0.85	1.55
Maternal job at birth's industry (NAF-2000 codes)	1.13	0.84	1.52
Paternal job at birth's industry (NAF-2000 codes)	1.11	0.82	1.50
Maternal social and occupational category at birth (PCS-2003)	1.12	0.83	1.50
Paternal social and occupational category at birth (PCS-2003)	1.11	0.83	1.50
Maternal education	1.10	0.82	1.49
Paternal incomes at birth	1.12	0.83	1.51
Urban status of the place of birth	1.13	0.83	1.53
French region of the birthplace	1.20	0.87	1.66
Domestic use of pesticides during early periods of development [b]	1.10	0.99	1.21
Domestic use of fungicides during early periods of development [b]	1.09	0.99	1.21

[a] Estimates obtained comparing TGCT cases to group A and group B controls combined. Analysis was restricted to subjects with no missing data for the exposure variable (1.8% excluded)

[b] As 50% of data were missing, multiple imputation by chain equation (MICE) was performed on these adjustment variables under the missing at random (MAR) assumption, using 25 datasets. Predictive variables were maternal education level, parental incomes at birth and the rural or urban status of the birthplace

Table S2. Continued from Table 1

	Group A controls (N=393)	Group B controls (N=290)	TGCT cases (N=472)
	n (%)	n (%)	n (%)
**PARENTAL OCCUPATIONS**			
Maternal social and occupational category at birth (PCS-2003)			
Farmers	3 (0.8)	5 (1.7)	5 (1.1)
Artisans,entrepreneurs	7 (1.8)	9 (3.1)	22 (4.7)
Executives and intellectual professions	26 (6.6)	35 (12.1)	32 (6.8)
Intermediate occupations	61 (15.5)	47 (16.2)	74 (15.7)
Employees	96 (24.4)	75 (25.9)	124 (26.3)
Workers	46 (11.7)	10 (3.4)	29 (6.1)
Not employed	151 (38.4)	108 (37.2)	185 (39.2)
Missing	3 (0.8)	1 (0.3)	1 (0.2)
Paternal social and occupational category at birth (PCS-2003)			
Farmers	12 (3.1)	7 (2.4)	21 (4.4)
Artisans,entrepreneurs	19 (4.8)	16 (5.5)	28 (5.9)
Executives and intellectual professions	57 (14.5)	60 (20.7)	69 (14.6)
Intermediate occupations	79 (20.1)	71 (24.5)	95 (20.1)
Employees	27 (6.9)	37 (12.8)	51 (10.8)
Workers	161 (41.0)	74 (25.5)	151 (32.0)
Not employed	34 (8.7)	19 (6.6)	51 (10.8)
Missing	4 (1.0)	6 (2.1)	6 (1.3)
Maternal educationa			
Baccalaureate's degree or less	83 (21.1)	59 (20.3)	192 (40.7)
Graduate studies	35 (8.9)	52 (17.9)	84 (17.8)
Other	26 (6.6)	18 (6.2)	28 (5.9)
Missing	249 (63.4)	161 (55.5)	168 (35.6)
Parental incomes at birtha			
0 to less than 5,000€	42 (10.7)	35 (12.1)	63 (13.3)
5,000 to less than 10,000€	22 (5.6)	18 (6.2)	55 (11.7)
10,000 to less than 20,000€	22 (5.6)	16 (5.5)	41 (8.7)
20,000 to less than 30,000€	5 (1.3)	13 (4.5)	21 (4.4)
30,000€ and more	5 (1.3)	5 (1.7)	24 (5.1)
Missing	297 (75.6)	203 (70.0)	268 (56.8)
**DOMESTIC USE OF PESTICIDES DURING EARLY PERIODS OF DEVELOPMENT**[a]			
No	29 (7.4)	28 (9.7)	62 (13.1)
Yes	114 (29.0)	101 (34.8)	241 (51.1)
Missing	250 (63.6)	161 (55.5)	169 (35.8)

a Missing data are due to the absence of mothers' interview (N=577)

Table S3. Odds ratios (OR) and 95% confidence intervals (CI) for GCNIS-related TGCT associated with GIS-based land use in a 500 m buffer around place of residence at birth, stratified analysis on birth cohort, case-control study, N=1155 France, 2015-2018

Birth cohort: 1970-1980

	Group A controls (N=138)		Group B controls (N=87)		All TGCT cases (N=139)				
	N	(%)	N	(%)	N	(%)	OR[a]	Lower 95% CI	Upper 95% CI
**Presence of arable lands, orchards and vineyards**									
No	45	(32.6)	30	(34.5)	38	(27.3)	1.00		
Yes	92	(66.7)	57	(65.5)	97	(69.8)	1.17	0.72	1.90
Missing	1	(0.7)	0	(0.0)	4	(2.9)	-	-	-
**Presence of arable lands**									
No	46	(33.3)	31	(35.6)	41	(29.5)	1.00		
Yes	91	(65.9)	56	(64.4)	94	(67.6)	1.10	0.68	1.77
Missing	1	(0.7)	0	(0.0)	4	(2.9)	-	-	-
**Presence of orchards**									
No	122	(88.4)	84	(96.6)	121	(87.1)	1.00		
Yes	15	(10.9)	3	(3.4)	14	(10.1)	1.29	0.61	2.72
Missing	1	(0.7)	0	(0.0)	4	(2.9)	-	-	-
**Presence of vineyards**									
No	121	(87.7)	82	(94.3)	124	(89.2)	1.00		
Yes	16	(11.6)	5	(5.7)	11	(7.9)	0.79	0.36	1.72
Missing	1	(0.7)	0	(0.0)	4	(2.9)	-	-	-
**Percentage of area of arable land use, tertiles (%)**									
[0.00-0.12]	47	(34.1)	32	(36.8)	45	(32.4)	1.00		
]0.12-11.44]	45	(32.6)	26	(29.9)	50	(36.0)	1.15	0.68	1.95
>11.44	45	(32.6)	29	(33.3)	40	(28.8)	0.84	0.48	1.45
Missing	1	(0.7)	0	(0.0)	4	(2.9)	-	-	-
**Percentage of area of land use, tertiles (%)**									
[0.00-0.30]	48	(34.8)	31	(35.6)	46	(33.1)	1.00		
]0.30-13.66]	45	(32.6)	25	(28.7)	49	(35.3)	1.13	0.67	1.92
>13.66	44	(31.9)	31	(35.6)	40	(28.8)	0.81	0.47	1.39
Missing	1	(0.7)	0	(0.0)	4	(2.9)	-	-	-

Birth cohort: 1981-1990

	Group A controls (N=220)		Group B controls (N=182)		All TGCT cases (N=256)				
	N	(%)	N	(%)	N	(%)	OR[a]	Lower 95% CI	Upper 95% CI
**Presence of arable lands, orchards and vineyards**									
No	46	(20.9)	55	(30.2)	60	(23.4)	1.00		
Yes	170	(77.3)	123	(67.6)	190	(74.2)	1.02	0.69	1.51
Missing	4	(1.8)	4	(2.2)	6	(2.3)	-	-	-
**Presence of arable lands**									
No	50	(22.7)	56	(30.8)	61	(23.8)	1.00		
Yes	166	(75.5)	122	(67.0)	189	(73.8)	1.07	0.73	1.57
Missing	4	(1.8)	4	(2.2)	6	(2.3)	-	-	-
**Presence of orchards**									
No	200	(90.9)	168	(92.3)	219	(85.5)	1.00		
Yes	16	(7.3)	10	(5.5)	31	(12.1)	2.02	1.16	3.52
Missing	4	(1.8)	4	(2.2)	6	(2.3)	-	-	-
**Presence of vineyards**									
No	191	(86.8)	172	(94.5)	226	(88.3)	1.00		
Yes	25	(11.4)	6	(3.3)	24	(9.4)	1.20	0.66	2.19
Missing	4	(1.8)	4	(2.2)	6	(2.3)	-	-	-
**Percentage of area of arable land use, tertiles (%)**									
[0.00-0.12]	57	(25.9)	60	(33.0)	67	(26.2)	1.00		
]0.12-11.44]	80	(36.4)	57	(31.3)	93	(36.3)	1.12	0.74	1.70
>11.44	79	(35.9)	61	(33.5)	90	(35.2)	1.08	0.71	1.64
Missing	4	(1.8)	4	(2.2)	6	(2.3)	-	-	-
**Percentage of area of land use, tertiles (%)**									
[0.00-0.30]	62	(28.2)	63	(34.6)	70	(27.3)	1.00		
]0.30-13.66]	77	(35.0)	58	(31.9)	86	(33.6)	1.09	0.72	1.64
>13.66	77	(35.0)	57	(31.3)	94	(36.7)	1.22	0.81	1.84
Missing	4	(1.8)	4	(2.2)	6	(2.3)	-	-	-

Birth cohort: 1991-1999

	Group A controls (N=35)		Group B controls (N=21)		All TGCT cases (N=77)					
	N	(%)	N	(%)	N	(%)	OR[a]	Lower 95% CI	Upper 95% CI	p-INT[b]
**Presence of arable lands, orchards and vineyards**										0.58
No	12	(34.3)	5	(23.8)	20	(26.0)	1.00			
Yes	22	(62.9)	16	(76.2)	56	(72.7)	1.25	0.53	2.91	
Missing	1	(2.9)	0	(0.0)	1	(1.3)	-	-	-	
**Presence of arable lands**										0.78
No	14	(40.0)	5	(23.8)	22	(28.6)	1.00			
Yes	20	(57.1)	16	(76.2)	54	(70.1)	1.24	0.54	2.84	
Missing	1	(2.9)	0	(0.0)	1	(1.3)	-	-	-	
**Presence of orchards**										0.32
No	31	(88.6)	17	(81.0)	72	(93.5)	1.00			
Yes	3	(8.6)	4	(19.0)	4	(5.2)	-	-	-	
Missing	1	(2.9)	0	(0.0)	1	(1.3)	-	-	-	
**Presence of vineyards**										0.35
No	28	(80.0)	15	(71.4)	69	(89.6)	1.00			
Yes	1	(2.9)	0	(0.0)	1	(1.3)	-	-	-	
Missing	6	(17.1)	6	(28.6)	7	(9.1)	-	-	-	
**Percentage of area of arable land use, tertiles (%)**										0.79
[0.00-0.12]	15	(42.9)	5	(23.8)	24	(31.2)	1.00			
]0.12-11.44]	11	(31.4)	9	(42.9)	29	(37.7)	1.28	0.53	3.12	
>11.44	8	(22.9)	7	(33.3)	23	(29.9)	1.21	0.48	3.08	
Missing	1	(2.9)	0	(0.0)	1	(1.3)	-	-	-	
**Percentage of area of land use, tertiles (%)**										0.63
[0.00-0.30]	15	(42.9)	5	(23.8)	23	(29.9)	1.00			
]0.30-13.66]	11	(31.4)	8	(38.1)	28	(36.4)	1.43	0.58	3.51	
>13.66	8	(22.9)	8	(38.1)	25	(32.5)	1.33	0.53	3.36	
Missing	1	(2.9)	0	(0.0)	1	(1.3)	-	-	-	

[a] Estimates obtained comparing TGCT cases to group A and group B controls combined. Analysis was restricted to subjects with no missing data for the exposure variable (1.8% excluded)

[b] P-value for interaction derived from the Likelihood Ratio Test comparing the models with and without interaction terms.

Table S4. Odds ratios (OR) and 95% confidence intervals (CI) for GCNIS-related TGCT associated with GIS-based land use in a 500 m buffer around place of residence at birth, stratified analyses on season of pregnancy, case-control study, N=1155, France, 2015-2018

	First trimester of pregnancy in Autumn/Winter									First trimester of pregnancy in Spring/Summer									
	Group A controls (N=102)		Group B controls (N=79)		All TGCT cases (N=127)					Group A controls (N=287)		Group B controls (N=207)		All TGCT cases (N=335)					
	N	(%)	N	(%)	N	(%)	OR[a]	Lower 95% CI	Upper 95% CI	N	(%)	N	(%)	N	(%)	OR[a]	Lower 95% CI	Upper 95% CI	p-INT [b]
**Presence of arable lands, orchards and vineyards**																			0.61
No	24	(23.5)	29	(36.7)	39	(30.7)	1.00			76	(26.5)	60	(29.0)	76	(22.7)	1.00			
Yes	76	(74.5)	49	(62.0)	85	(66.9)	1.01	0.57	1.81	207	(72.1)	144	(69.6)	252	(75.2)	1.27	0.89	1.81	
Missing	2	(2.0)	1	(1.3)	3	(2.4)	-	-	-	4	(1.4)	3	(1.4)	7	(2.1)	-	-	-	
**Presence of arable lands**																			0.63
No	25	(24.5)	29	(36.7)	39	(30.7)	1.00			82	(28.6)	62	(30.0)	82	(24.5)	1.00			
Yes	75	(73.5)	49	(62.0)	85	(66.9)	1.04	0.58	1.86	201	(70.0)	142	(68.6)	246	(73.4)	1.28	0.91	1.82	
Missing	2	(2.0)	1	(1.3)	3	(2.4)	-	-	-	4	(1.4)	3	(1.4)	7	(2.1)	-	-	-	
**Presence of orchards**																			0.03
No	91	(89.2)	73	(92.4)	116	(91.3)	1.00			258	(89.9)	192	(92.8)	290	(86.6)	1.00			
Yes	9	(8.8)	5	(6.3)	8	(6.3)	0.82	0.31	2.16	25	(8.7)	12	(5.8)	38	(11.3)	1.70	1.03	2.82	
Missing	2	(2.0)	1	(1.3)	3	(2.4)	-	-	-	4	(1.4)	3	(1.4)	7	(2.1)	-	-	-	
**Presence of vineyards**																			0.21
No	87	(85.3)	74	(93.7)	115	(90.6)	1.00			249	(86.8)	191	(92.3)	297	(88.7)	1.00			
Yes	13	(12.7)	4	(5.1)	9	(7.1)	0.69	0.27	1.79	34	(11.8)	13	(6.3)	31	(9.3)	0.90	0.53	1.52	
Missing	2	(2.0)	1	(1.3)	3	(2.4)	-	-	-	4	(1.4)	3	(1.4)	7	(2.1)	-	-	-	
**Percentage of area of arable land use, tertiles (%)**																			0.39
[0.00-0.12]	27	(26.5)	30	(38.0)	42	(33.1)	1.00			89	(31.0)	66	(31.9)	91	(27.2)	1.00			
]0.12-11.44]	37	(36.3)	19	(24.1)	44	(34.6)	1.13	0.61	2.10	99	(34.5)	73	(35.3)	124	(37.0)	1.25	0.86	1.82	
>11.44	36	(35.3)	29	(36.7)	38	(29.9)	0.85	0.43	1.67	95	(33.1)	65	(31.4)	113	(33.7)	1.19	0.81	1.75	
Missing	2	(2.0)	1	(1.3)	3	(2.4)	-	-	-	4	(1.4)	3	(1.4)	7	(2.1)	-	-	-	
**Percentage of area of land use, tertiles (%)**																			0.34
[0.00-0.30]	29	(28.4)	30	(38.0)	44	(34.6)	1.00			93	(32.4)	68	(32.9)	92	(27.5)	1.00			
]0.30-13.66]	36	(35.3)	18	(22.8)	40	(31.5)	1.09	0.57	2.08	97	(33.8)	73	(35.3)	119	(35.5)	1.21	0.84	1.76	
>13.66	35	(34.3)	30	(38.0)	40	(31.5)	0.84	0.44	1.60	93	(32.4)	63	(30.4)	117	(34.9)	1.29	0.88	1.87	
Missing	2	(2.0)	1	(1.3)	3	(2.4)	-	-	-	4	(1.4)	3	(1.4)	7	(2.1)	-	-	-	

[a] Estimates obtained comparing TGCT cases to group A and group B controls combined. Analysis was restricted to subjects with no missing data for the exposure variable (1.8% excluded)

[b] P-value for interaction derived from the Likelihood Ratio Test comparing the models with and without interaction terms.

Table S5. Odds ratios (OR) and 95% confidence intervals (CI) for GCNIS-related TGCT associated with GIS-based land use in a 500 m buffer around place of residence at birth, stratified analyses on birthplace status, case-control study, N=1155, France, 2015-2018

	**Rural birthplace**									**Urban birthplace**									
	Group A controls (N=99)		Group B controls (N=58)		All TGCT cases (N=120)					Group A controls (N=294)		Group B controls (N=232)		All TGCT cases (N=252)					
	N	(%)	N	(%)	N	(%)	OR[a]	Lower 95% CI	Upper 95% CI	N	(%)	N	(%)	N	(%)	OR[a]	Lower 95% CI	Upper 95% CI	p-INT [b]
**Presence of arable lands, orchards and vineyards**																			0.81
No	4	(4.0)	0	(0.0)	4	(3.3)	1.00			99	(33.7)	90	(38.8)	114	(32.4)	1.00			
Yes	94	(94.9)	58	(100.0)	114	(95.0)	-	-	-	190	(64.6)	138	(59.5)	229	(65.1)	1.21	0.88	1.66	
Missing	1	(1.0)	0	(0.0)	2	(1.7)	-	-	-	5	(1.7)	4	(1.7)	9	(2.6)	-	-	-	
**Presence of arable lands**																			1.00
No	5	(5.1)	0	(0.0)	4	(3.3)	1.00			105	(35.7)	92	(39.7)	120	(34.1)	1.00			
Yes	93	(93.9)	58	(100.0)	114	(95.0)	-	-	-	184	(62.6)	136	(58.6)	223	(63.4)	1.22	0.89	1.67	
Missing	1	(1.0)	0	(0.0)	2	(1.7)	-	-	-	5	(1.7)	4	(1.7)	9	(2.6)	-	-	-	
**Presence of orchards**																			0.30
No	83	(83.8)	56	(96.6)	97	(80.8)	1.00			270	(91.8)	213	(91.8)	315	(89.5)	1.00			
Yes	15	(15.2)	2	(3.4)	21	(17.5)	1.94	0.87	4.34	19	(6.5)	15	(6.5)	28	(8.0)	1.29	0.75	2.21	
Missing	1	(1.0)	0	(0.0)	2	(1.7)	-	-	-	5	(1.7)	4	(1.7)	9	(2.6)	-	-	-	
**Presence of vineyards**																			0.34
No	83	(83.8)	54	(93.1)	101	(84.2)	1.00			257	(87.4)	215	(92.7)	318	(90.3)	1.00			
Yes	15	(15.2)	4	(6.9)	17	(14.2)	0.91	0.37	2.26	32	(10.9)	13	(5.6)	25	(7.1)	0.76	0.44	1.30	
Missing	1	(1.0)	0	(0.0)	2	(1.7)	-	-	-	5	(1.7)	4	(1.7)	9	(2.6)	-	-	-	
**Percentage of area of arable land use, tertiles (%)**																			0.87
[0.00-0.12]	7	(7.1)	1	(1.7)	5	(4.2)	1.00			112	(38.1)	96	(41.4)	131	(37.2)	1.00			
]0.12-11.44]	19	(19.2)	8	(13.8)	25	(20.8)	1.69	0.37	7.64	117	(39.8)	84	(36.2)	147	(41.8)	1.21	0.87	1.69	
>11.44	72	(72.7)	49	(84.5)	88	(73.3)	1.62	0.38	6.87	60	(20.4)	48	(20.7)	65	(18.5)	1.03	0.69	1.54	
Missing	1	(1.0)	0	(0.0)	2	(1.7)	-	-	-	5	(1.7)	4	(1.7)	9	(2.6)	-	-	-	
**Percentage of area of land use, tertiles (%)**																			0.99
[0.00-0.30]	6	(6.1)	0	(0.0)	5	(4.2)	1.00			119	(40.5)	99	(42.7)	134	(38.1)	1.00			
]0.30-13.66]	17	(17.2)	8	(13.8)	21	(17.5)	0.82	0.16	4.15	116	(39.5)	83	(35.8)	142	(40.3)	1.19	0.86	1.64	
>13.66	75	(75.8)	50	(86.2)	92	(76.7)	0.78	0.17	3.64	54	(18.4)	46	(19.8)	67	(19.0)	1.16	0.78	1.73	
Missing	1	(1.0)	0	(0.0)	2	(1.7)	-	-	-	5	(1.7)	4	(1.7)	9	(2.6)	-	-	-	

[a] Estimates obtained comparing TGCT cases to group A and group B controls combined. Analysis was restricted to subjects with no missing data for the exposure variable (1.8% excluded)

[b] P-value for interaction derived from the Likelihood Ratio Test comparing the models with and without interaction terms.

Table S6. Odds ratios (OR) and 95% confidence intervals (CI) for GCNIS-related TGCT associated with GIS-based land use in a 500 m buffer around place of residence at birth, stratified analyses on geocoding accuracy, case-control study, N=1155, France, 2015-2018

	Less accurate geocoding									Accurate geocoding									
	Group A controls (N=105)		Group B controls (N=76)		All TGCT cases (N=119)					Group A controls (N=288)		Group B controls (N=214)		All TGCT cases (N=353)					
	N	(%)	N	(%)	N	(%)	OR[a]	Lower 95% CI	Upper 95% CI	N	(%)	N	(%)	N	(%)	OR[a]	Lower 95% CI	Upper 95% CI	p-INT [b]
**Presence of arable lands, orchards and vineyards**																			0.08
No	25	(23.8)	17	(22.4)	17	(14.3)	1.00			78	(27.1)	73	(34.1)	101	(28.6)	1.00			
Yes	77	(73.3)	57	(75.0)	100	(84.0)	1.66	0.84	3.26	207	(71.9)	139	(65.0)	243	(68.8)	1.05	0.75	1.47	
Missing	3	(2.9)	2	(2.6)	2	(1.7)	-	-	-	3	(1.0)	2	(0.9)	9	(2.5)	-	-	-	
**Presence of arable lands**																			0.26
No	25	(23.8)	18	(23.7)	21	(17.6)	1.00			85	(29.5)	74	(34.6)	103	(29.2)	1.00			
Yes	77	(73.3)	56	(73.7)	96	(80.7)	1.35	0.71	2.56	200	(69.4)	138	(64.5)	241	(68.3)	1.10	0.79	1.54	
Missing	3	(2.9)	2	(2.6)	2	(1.7)	-	-	-	3	(1.0)	2	(0.9)	9	(2.5)	-	-	-	
**Presence of orchards**																			0.82
No	88	(83.8)	69	(90.8)	102	(85.7)	1.00			265	(92.0)	200	(93.5)	310	(87.8)	1.00			
Yes	14	(13.3)	5	(6.6)	15	(12.6)	1.43	0.64	3.20	20	(6.9)	12	(5.6)	34	(9.6)	1.53	0.90	2.60	
Missing	3	(2.9)	2	(2.6)	2	(1.7)	-	-	-	3	(1.0)	2	(0.9)	9	(2.5)	-	-	-	
**Presence of vineyards**																			1.00
No	90	(85.7)	67	(88.2)	104	(87.4)	1.00			250	(86.8)	202	(94.4)	315	(89.2)	1.00			
Yes	12	(11.4)	7	(9.2)	13	(10.9)	0.64	0.25	1.64	35	(12.2)	10	(4.7)	29	(8.2)	0.91	0.53	1.56	
Missing	3	(2.9)	2	(2.6)	2	(1.7)	-	-	-	3	(1.0)	2	(0.9)	9	(2.5)	-	-	-	
**Percentage of area of arable land use, tertiles (%)**																			0.47
[0.00-0.12]	28	(26.7)	20	(26.3)	25	(21.0)	1.00			91	(31.6)	77	(36.0)	111	(31.4)	1.00			
]0.12-11.44]	29	(27.6)	23	(30.3)	42	(35.3)	1.67	0.82	3.40	107	(37.2)	69	(32.2)	130	(36.8)	1.15	0.80	1.65	
>11.44	45	(42.9)	31	(40.8)	50	(42.0)	1.17	0.60	2.26	87	(30.2)	66	(30.8)	103	(29.2)	1.01	0.69	1.48	
Missing	3	(2.9)	2	(2.6)	2	(1.7)	-	-	-	3	(1.0)	2	(0.9)	9	(2.5)	-	-	-	
**Percentage of area of land use, tertiles (%)**																			0.62
[0.00-0.30]	29	(27.6)	18	(23.7)	24	(20.2)	1.00			96	(33.3)	81	(37.9)	115	(32.6)	1.00			
]0.30-13.66]	28	(26.7)	27	(35.5)	39	(32.8)	1.50	0.74	3.05	105	(36.5)	64	(29.9)	124	(35.1)	1.14	0.80	1.63	
>13.66	45	(42.9)	29	(38.2)	54	(45.4)	1.30	0.67	2.53	84	(29.2)	67	(31.3)	105	(29.7)	1.05	0.72	1.53	
Missing	3	(2.9)	2	(2.6)	2	(1.7)	-	-	-	3	(1.0)	2	(0.9)	9	(2.5)	-	-	-	

[a] Estimates obtained comparing TGCT cases to group A and group B controls combined. Analysis was restricted to subjects with no missing data for the exposure variable (1.8% excluded)

[b] P-value for interaction derived from the Likelihood Ratio Test comparing the models with and without interaction terms.

Table S7. Odds ratios (OR) and 95% confidence intervals (CI) for GCNIS-related TGCT associated with GIS-based land use in a 500 m buffer around place of residence at birth, sensitivity analyses, case-control study, France, 2015-2018

Excluding cases with personal history of cryptorchidism, N=1115

	Group A controls (N=393)		Group B controls (N=290)		TGCT cases (N=432)				
	N	(%)	N	(%)	N	(%)	OR[a]	Lower 95% CI	Upper 95% CI
**Presence of arable lands, orchards and vineyards**									
No	103	(26.2)	90	(31.0)	110	(25.5)	1.00		
Yes	284	(72.3)	196	(67.6)	311	(72.0)	1.11	0.83	1.50
Missing	6	(1.5)	4	(1.4)	11	(2.5)	-	-	-
**Presence of arable lands**									
No	110	(28.0)	92	(31.7)	116	(26.9)	1.00		
Yes	277	(70.5)	194	(66.9)	305	(70.6)	1.12	0.83	1.49
Missing	6	(1.5)	4	(1.4)	11	(2.5)	-	-	-
**Presence of orchards**									
No	353	(89.8)	269	(92.8)	376	(87.0)	1.00		
Yes	34	(8.7)	17	(5.9)	45	(10.4)	1.57	1.01	2.44
Missing	6	(1.5)	4	(1.4)	11	(2.5)	-	-	-
**Presence of vineyards**									
No	340	(86.5)	269	(92.8)	382	(88.4)	1.00		
Yes	47	(12.0)	17	(5.9)	39	(9.0)	0.91	0.58	1.44
Missing	6	(1.5)	4	(1.4)	11	(2.5)	-	-	-
**Percentage of area of arable land use, tertiles (%)**									
[0.00-0.12]	119	(30.3)	97	(33.4)	126	(29.2)	1.00		
]0.12-11.44]	136	(34.6)	92	(31.7)	156	(36.1)	1.17	0.85	1.62
>11.44	132	(33.6)	97	(33.4)	139	(32.2)	1.01	0.73	1.40
Missing	6	(1.5)	4	(1.4)	11	(2.5)	-	-	-
**Percentage of area of land use, tertiles (%)**									
[0.00-0.30]	125	(31.8)	99	(34.1)	129	(29.9)	1.00		
]0.30-13.66]	133	(33.8)	91	(31.4)	148	(34.3)	1.11	0.81	1.53
>13.66	129	(32.8)	96	(33.1)	144	(33.3)	1.06	0.77	1.47
Missing	6	(1.5)	4	(1.4)	11	(2.5)	-	-	-

Excluding cases and controls with family history of cryptorchidism and testicular cancer, N=1066

	Group A controls (N=377)		Group B controls (N=273)		TGCT cases (N=416)				
	N	(%)	N	(%)	N	(%)	OR[a]	Lower 95% CI	Upper 95% CI
**Presence of arable lands, orchards and vineyards**									
No	99	(26.3)	86	(31.5)	104	(25.0)	1.00		
Yes	273	(72.4)	183	(67.0)	301	(72.4)	1.17	0.86	1.58
Missing	5	(1.3)	4	(1.5)	11	(2.6)	-	-	-
**Presence of arable lands**									
No	106	(28.1)	88	(32.2)	110	(26.4)	1.00		
Yes	266	(70.6)	181	(66.3)	295	(70.9)	1.17	0.86	1.57
Missing	5	(1.3)	4	(1.5)	11	(2.6)	-	-	-
**Presence of orchards**									
No	341	(90.5)	252	(92.3)	360	(86.5)	1.00		
Yes	31	(8.2)	17	(6.2)	45	(10.8)	1.59	1.02	2.48
Missing	5	(1.3)	4	(1.5)	11	(2.6)	-	-	-
**Presence of vineyards**									
No	325	(86.2)	254	(93.0)	368	(88.5)	1.00		
Yes	47	(12.5)	15	(5.5)	37	(8.9)	0.85	0.54	1.35
Missing	5	(1.3)	4	(1.5)	11	(2.6)	-	-	-
**Percentage of area of arable land use, tertiles (%)**									
[0.00-0.12]	115	(30.5)	93	(34.1)	120	(28.8)	1.00		
]0.12-11.44]	129	(34.2)	86	(31.5)	150	(36.1)	1.20	0.87	1.67
>11.44	128	(34.0)	90	(33.0)	135	(32.5)	1.07	0.77	1.50
Missing	5	(1.3)	4	(1.5)	11	(2.6)	-	-	-
**Percentage of area of land use, tertiles (%)**									
[0.00-0.30]	120	(31.8)	95	(34.8)	121	(29.1)	1.00		
]0.30-13.66]	128	(34.0)	85	(31.1)	142	(34.1)	1.16	0.84	1.62
>13.66	124	(32.9)	89	(32.6)	142	(34.1)	1.17	0.84	1.63
Missing	5	(1.3)	4	(1.5)	11	(2.6)	-	-	-

Excluding TGCT cases not confirmed by pathology reports, N=1112

	Group A controls (N=393)		Group B controls (N=290)		TGCT cases (N=429)				
	N	(%)	N	(%)	N	(%)	OR[a]	Lower 95% CI	Upper 95% CI
**Presence of arable lands, orchards and vineyards**									
No	103	(26.2)	90	(31.0)	107	(24.9)	1.00		
Yes	284	(72.3)	196	(67.6)	312	(72.7)	1.11	0.82	1.50
Missing	6	(1.5)	4	(1.4)	10	(2.3)	-	-	-
**Presence of arable lands**									
No	110	(28.0)	92	(31.7)	112	(26.1)	1.00		
Yes	277	(70.5)	194	(66.9)	307	(71.6)	1.13	0.84	1.52
Missing	6	(1.5)	4	(1.4)	10	(2.3)	-	-	-
**Presence of orchards**									
No	353	(89.8)	269	(92.8)	372	(86.7)	1.00		
Yes	34	(8.7)	17	(5.9)	47	(11.0)	1.61	1.05	2.49
Missing	6	(1.5)	4	(1.4)	10	(2.3)	-	-	-
**Presence of vineyards**									
No	340	(86.5)	269	(92.8)	380	(88.6)	1.00		
Yes	47	(12.0)	17	(5.9)	39	(9.1)	0.89	0.56	1.39
Missing	6	(1.5)	4	(1.4)	10	(2.3)	-	-	-
**Percentage of area of arable land use, tertiles (%)**									
[0.00-0.12]	119	(30.3)	97	(33.4)	122	(28.4)	1.00		
]0.12-11.44]	136	(34.6)	92	(31.7)	156	(36.4)	1.17	0.85	1.61
>11.44	132	(33.6)	97	(33.4)	141	(32.9)	1.04	0.75	1.45
Missing	6	(1.5)	4	(1.4)	10	(2.3)	-	-	-
**Percentage of area of land use, tertiles (%)**									
[0.00-0.30]	125	(31.8)	99	(34.1)	125	(29.1)	1.00		
]0.30-13.66]	133	(33.8)	91	(31.4)	148	(34.5)	1.13	0.82	1.55
>13.66	129	(32.8)	96	(33.1)	146	(34.0)	1.10	0.80	1.52
Missing	6	(1.5)	4	(1.4)	10	(2.3)	-	-	-

[a] Estimates obtained comparing TGCT cases to group A and group B controls combined. Analysis was restricted to subjects with no missing data for the exposure variable (1.8% excluded)

Table S8. Odds ratios (OR) and 95% confidence intervals (CI) for GCNIS-related TGCT associated with GIS-based land use in a 1000 m buffer around place of residence at birth, sensitivity analyses, case-control study, N=1155, France, 2015-2018

	Total (N=1155)		Group A controls (N=393)		Group B controls (N=290)		All TGCT cases (N=472)					Non-Seminomas (N=203)					Seminomas (N=225)					p-HET [b]
	N	(%)	N	(%)	N	(%)	N	(%)	OR[a]	Lower 95% CI	Upper 95% CI	N	(%)	OR[a]	Lower 95% CI	Upper 95% CI	N	(%)	OR[a]	Lower 95% CI	Upper 95% CI	
**Presence of arable lands, orchards and vineyards**																						0.84
No	267	(23.1)	87	(22.1)	83	(28.6)	97	(20.6)	1.00			43	(21.2)	1.00			44	(19.6)	1.00			
Yes	864	(74.8)	300	(76.3)	201	(69.3)	363	(76.9)	1.24	0.91	1.67	156	(76.8)	1.20	0.78	1.84	174	(77.3)	1.27	0.85	1.90	
Missing	24	(2.1)	6	(1.5)	6	(2.1)	12	(2.5)	-	-	-	4	(2.0)	-	-	-	7	(3.1)	-	-	-	
**Presence of arable lands**																						0.72
No	276	(23.9)	92	(23.4)	84	(29.0)	100	(21.2)	1.00			44	(21.7)	1.00			45	(20.0)	1.00			
Yes	855	(74.0)	295	(75.1)	200	(69.0)	360	(76.3)	1.25	0.92	1.69	155	(76.4)	1.19	0.78	1.83	173	(76.9)	1.33	0.89	1.98	
Missing	24	(2.1)	6	(1.5)	6	(2.1)	12	(2.5)	-	-	-	4	(2.0)	-	-	-	7	(3.1)	-	-	-	
**Presence of orchards**																						0.95
No	999	(86.5)	341	(86.8)	258	(89.0)	400	(84.7)	1.00			174	(85.7)	1.00			186	(82.7)	1.00			
Yes	132	(11.4)	46	(11.7)	26	(9.0)	60	(12.7)	1.21	0.83	1.77	25	(12.3)	1.30	0.76	2.21	32	(14.2)	1.33	0.83	2.12	
Missing	24	(2.1)	6	(1.5)	6	(2.1)	12	(2.5)	-	-	-	4	(2.0)	-	-	-	7	(3.1)	-	-	-	
**Presence of vineyards**																						0.83
No	986	(85.4)	322	(81.9)	261	(90.0)	403	(85.4)	1.00			175	(86.2)	1.00			190	(84.4)	1.00			
Yes	145	(12.6)	65	(16.5)	23	(7.9)	57	(12.1)	0.87	0.59	1.28	24	(11.8)	0.83	0.48	1.42	28	(12.4)	0.90	0.55	1.47	
Missing	24	(2.1)	6	(1.5)	6	(2.1)	12	(2.5)	-	-	-	4	(2.0)	-	-	-	7	(3.1)	-	-	-	
**Percentage of area of arable land use, tertiles (%)**																						0.87
[0.00-0.50]	342	(29.6)	118	(30.0)	99	(34.1)	125	(26.5)	1.00			53	(26.1)	1.00			57	(25.3)	1.00			
]0.50-18.32]	407	(35.2)	141	(35.9)	85	(29.3)	181	(38.3)	1.34	0.98	1.84	78	(38.4)	1.30	0.83	2.03	89	(39.6)	1.48	0.98	2.23	
>18.32	382	(33.1)	128	(32.6)	100	(34.5)	154	(32.6)	1.15	0.84	1.59	68	(33.5)	1.22	0.77	1.93	72	(32.0)	1.22	0.80	1.86	
Missing	24	(2.1)	6	(1.5)	6	(2.1)	12	(2.5)	-	-	-	4	(2.0)	-	-	-	7	(3.1)	-	-	-	
**Percentage of area of land use, tertiles (%)**																						0.95
[0.00-0.94]	351	(30.4)	120	(30.5)	103	(35.5)	128	(27.1)	1.00			54	(26.6)	1.00			59	(26.2)	1.00			
]0.94-20.64]	393	(34.0)	140	(35.6)	83	(28.6)	170	(36.0)	1.28	0.93	1.75	74	(36.5)	1.29	0.83	2.00	83	(36.9)	1.39	0.93	2.09	
>20.64	387	(33.5)	127	(32.3)	98	(33.8)	162	(34.3)	1.21	0.88	1.67	71	(35.0)	1.27	0.81	1.98	76	(33.8)	1.27	0.84	1.93	
Missing	24	(2.1)	6	(1.5)	6	(2.1)	12	(2.5)	-	-	-	4	(2.0)	-	-	-	7	(3.1)	-	-	-	

[a] Estimates obtained comparing TGCT cases to group A and group B controls combined. Analysis was

[b] P-value for heterogeneity derived from the Likelihood Ratio Test, comparing seminoma versus non-seminoma tumors

Figure S1. Flowchart of the selection of the study population, TESTIS, France, 2015-2018.

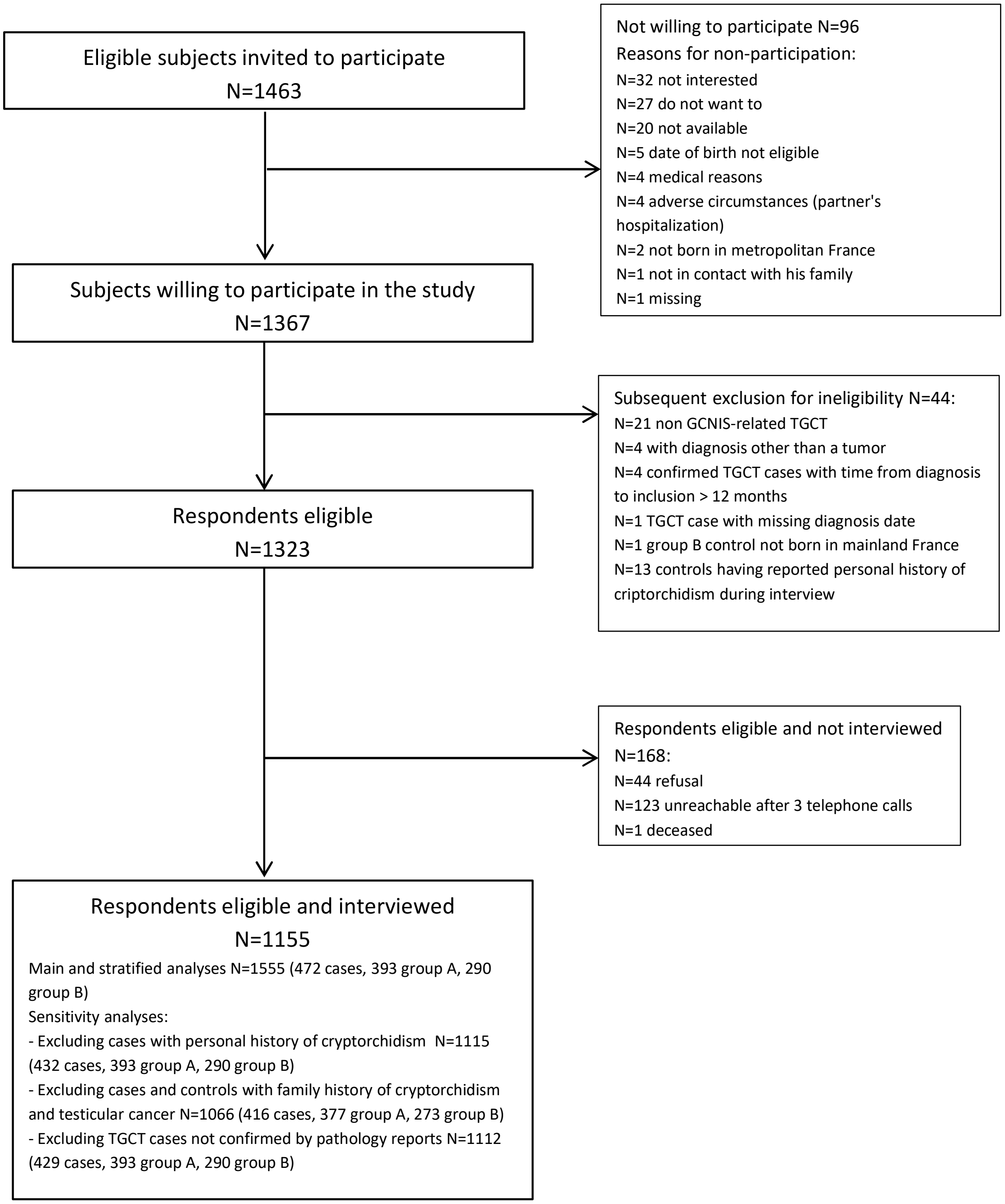

Figure S2. Illustration of the classification of land-use using the GOURAMIC® software in buffers around address of residence, TESTIS, France.

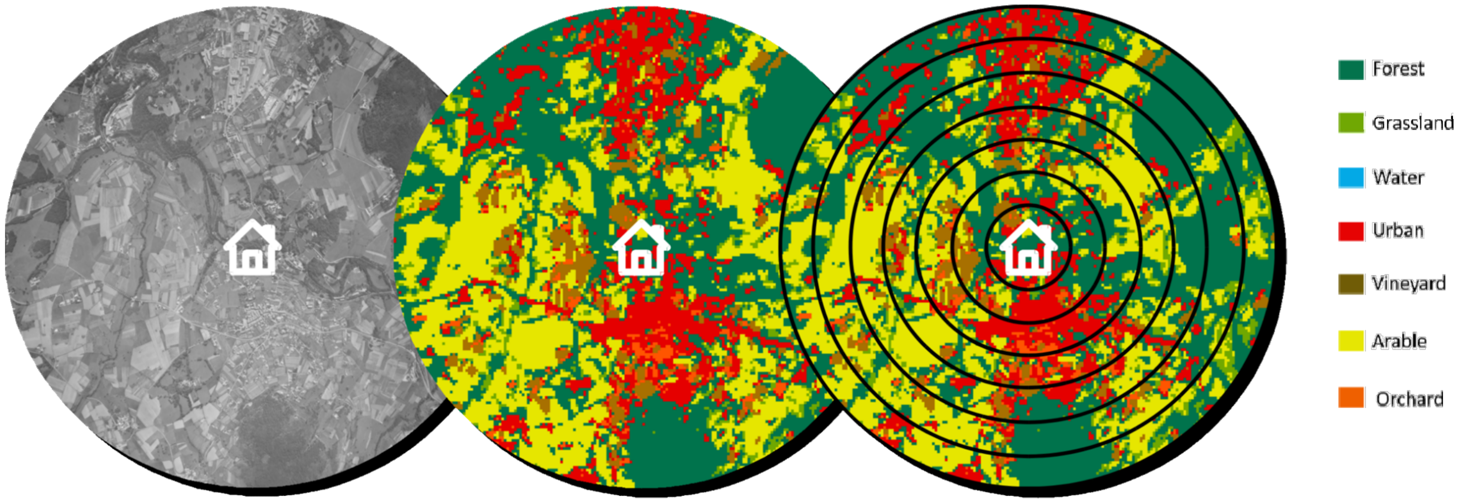

Supplement: Supplementary file 1 — Table S1: Odds ratios (OR) and 95% confidence intervals (CI) for GCNIS‐related TGCT associated with presence of arable lands, orchards and vineyards in a 500 m buffer around residence at birth, change in estimates, case–control study, N = 1155, France, 2015–2018. Table S2: Continued from Table 1. Table S3: Odds ratios (OR) and 95% confidence intervals (CI) for GCNIS‐related TGCT associated with GIS‐based land use in a 500 m buffer around place of residence at birth, stratified analysis on birth cohort, case–control study, N = 1155 France, 2015–2018. Table S4: Odds ratios (OR) and 95% confidence intervals (CI) for GCNIS‐related TGCT associated with GIS‐based land use in a 500 m buffer around place of residence at birth, stratified analyses on season of pregnancy, case–control study, N = 1155, France, 2015–2018. Table S5: Odds ratios (OR) and 95% confidence intervals (CI) for GCNIS‐related TGCT associated with GIS‐based land use in a 500 m buffer around place of residence at birth, stratified analyses on birthplace status, case–control study, N = 1155, France, 2015–2018. Table S6: Odds ratios (OR) and 95% confidence intervals (CI) for GCNIS‐related TGCT associated with GIS‐based land use in a 500 m buffer around place of residence at birth, stratified analyses on geocoding accuracy, case–control study, N = 1155, France, 2015–2018. Table S7: Odds ratios (OR) and 95% confidence intervals (CI) for GCNIS‐related TGCT associated with GIS‐based land use in a 500 m buffer around place of residence at birth, sensitivity analyses, case–control study, France, 2015–2018. Table S8: Odds ratios (OR) and 95% confidence intervals (CI) for GCNIS‐related TGCT associated with GIS‐based land use in a 1000 m buffer around place of residence at birth, sensitivity analyses, case–control study, N = 1155, France, 2015–2018. Figure S1: Flowchart of the selection of the study population, TESTIS, France, 2015–2018. Figure S2: Illustration of the classification of land‐use using the GOURAMIC sof [file IJC-159-1153-s001.pdf]
